# C-reactive protein as an early biomarker for malaria infection and monitoring of malaria severity: a meta-analysis

**DOI:** 10.1038/s41598-021-01556-0

**Published:** 2021-11-11

**Authors:** Polrat Wilairatana, Praphassorn Mahannop, Thanita Tussato, I-mee Hayeedoloh, Rachasak Boonhok, Wiyada Kwanhian Klangbud, Wanida Mala, Kwuntida Uthaisar Kotepui, Manas Kotepui

**Affiliations:** 1 Department of Clinical Tropical Medicine, Faculty of Tropical Medicine, Mahidol University, Bangkok, Thailand; 2 Medical Technology, School of Allied Health Sciences, Walailak University, Tha Sala, Nakhon Si Thammarat, Thailand

**Keywords:** Diagnostic markers, Prognostic markers

## Abstract

This study investigated whether C-reactive protein (CRP) can be used as a marker for the early detection and monitoring of malaria severity. Potentially relevant studies were searched in Medline (PubMed), Scopus, and Web of Science. Differences in CRP between (1) severe malaria and uncomplicated malaria, (2) uncomplicated malaria and asymptomatic malaria, (3) uncomplicated malaria and febrile/healthy controls, and (4) asymptomatic malaria and febrile/healthy controls were estimated using random-effects models. Twenty-nine studies were included for meta-analysis. The results of meta-analysis demonstrated higher mean CRP levels in (1) patients with severe malaria compared with uncomplicated malaria (*p* < 0.001, standard mean difference [SMD]: 1.52, 95% confidence interval [CI]: 0.91–2.12, I^2^: 95.1%), (2) patients with uncomplicated malaria than in those with asymptomatic malaria (*p*: 0.001, SMD: 1.65, 95% CI: 0.67–2.62, I^2^: 96.7%), (3) patients with uncomplicated malaria compared with febrile/healthy controls (*p* < 0.001, SMD: 2.38, 95% CI: 1.37–3.40, I^2^: 98.5%), and (4) patients with asymptomatic malaria compared with febrile/healthy controls (*p* < 0.001, SMD: 2.55, 95% CI: 1.60–3.50, I^2^: 99.2%). This study demonstrated CRP levels are a biomarker for the early detection and monitoring of malaria severity.

## Introduction

Malaria remains an ongoing public health problem in 87 malaria-endemic countries^1^. Globally, the World Health Organization (WHO) estimates that 229 million people have been infected with *Plasmodium* spp., and Nigeria, the Democratic Republic of the Congo, Uganda, Mozambique, and Niger accounted for about 51% of all malaria cases globally in 2019^1^. In malaria-endemic areas, the presence of fever is a classical symptom of malaria; however, fever is also caused by other infections including bacterial or viral infection. Therefore, the WHO recommends that microscopy or rapid diagnostic test (RDT) is a requirement for malaria diagnosis before treatment in all febrile individuals^2^. In situations where microscopy or RDT is unavailable, some biomarkers could provide an alternative method for malaria diagnosis. Some blood biomarkers have been well-described as candidates for malaria infection, including decreases in leukocytes or platelet count^3,4^. Moreover, other markers related to the immune response to malaria infection have also been assessed, including acute-phase proteins. The acute-phase protein is a nonspecific protein released during infection, tissue damage, tissue injury, and the inflammation process. C-reactive protein (CRP), which is one of the acute-phase proteins, is a classic marker for inflammation and is synthesized by the liver cells. The synthesis of CRP is modulated mainly by IL-6, TNF-α and IFN-γ. Previous studies demonstrated that these proteins were released during malaria infection^5–8^. A previous study also demonstrated a strong binding of CRP with infected erythrocytes that activate the complement pathway^9^ leading to erythrocyte clearance and hemolysis, which is the causative factor of anemia, one of the severe manifestations of malaria^9^. Additionally, CRP can initiate phagocytosis of the dying or dead cells by binding to phosphocholine and phosphoethanolamine on the surface of dying or dead cells^10^.

Previous studies showed that CRP could be used as a predictor of chronic diseases including diabetes and cancer^11–13^. Additionally, CRP was used as a biomarker for neonatal sepsis^14^ and bacterial pneumonia^15^, thus differentiating malaria from bacterial infection^16^. A previous study conducted in China demonstrated that the CRP/albumin ratio might predict the severity of neonatal sepsis^17^ and severe burn^18^. A recent review reported that CRP and procalcitonin could be used as point-of-care biomarkers to guide antibiotic prescription for acute febrile illness^19^. Interestingly, during low-grade inflammation without infection, serum CRP levels between 1 and 10 mg/L were associated with an increased risk of cardiovascular disease^20^. The high-sensitivity methods used to detect very low amounts of serum CRP were referred to as high-sensitivity CRP (hs-CRP)^20^. Because CRP is usually measured using a rapid, simple, nonspecific, routine laboratory test, CRP levels might be useful in assisting with the early detection of malaria and determining malarial severity.

The most recent meta-analysis proposed that CRP could be a candidate marker to distinguish bacterial from nonbacterial infections^21^. However, the meta-analysis demonstrating the possibility of CRP as an early marker for malaria infection and malaria severity has not been investigated. Therefore, this study aimed to determine the possibility of CRP being used as a candidate marker for the early detection and monitoring of malaria severity by analyzing the difference in CRP levels between patients with severe, uncomplicated, and asymptomatic malaria in comparison to febrile or healthy controls.

## Methods

### Protocol and registrations

The protocol of systematic review and meta-analysis was registered at The International Prospective Register of Systematic Reviews (PROSPERO) ID: CRD42021244441. The reporting of systematic review and meta-analysis followed the Preferred Reporting Items for Systematic Reviews and Meta-analyses (PRISMA)^22^.

### Malaria definitions

The clinical symptoms of patients who were infected with *Plasmodium* spp. were categorized into three groups: severe malaria, uncomplicated malaria, and asymptomatic malaria. Severe malaria is defined as the presence of malaria parasites with one or more of the complications listed in WHO criteria for severe malaria^2^. Uncomplicated malaria is defined as patients who developed symptoms of malaria with the presence of malaria parasites but no features of severe malaria. Asymptomatic malaria is defined as the presence of malaria parasites in the blood without any symptoms of malaria.

### Comparator/control group definitions

Comparator/control groups were divided into febrile control and healthy control groups. Febrile controls were patients with a non-malarial febrile illness who visited hospitals with fever and suspected malaria but with a microscopic examination that demonstrated no parasites. Healthy controls were participants who lived in the same area as patients with malaria.

### Search strategy

Potentially relevant studies were searched in three databases, namely, PubMed, Scopus, and ISI Web of Sciences, using the search terms (“C-reactive protein” OR “C-reactive protein” OR “CRP” OR “hs-CRP” OR “hs-CRP” AND “malaria” OR “Plasmodium”). The searches were ended on January 26, 2021. The details of the search terms and search strategy are provided in Table S1.

### Eligibility criteria

The inclusion criteria of this study were (1) prospective or retrospective observational study, cohort, or case–control studies that reported quantitative CRP measurements from malaria patients and (2) study must be in the English language. The exclusion criteria included the following: animal studies, book or book series, case study or case series, clinical drug trials without CRP at baseline, the study whose data were not extracted, editorial, letters to the editor, comments, short reports, experimental or genetic study of CRP, studies that reported the CRP with less than 10 patients, no full text, quiz, reviews or systematic review, and study using the same participants.

### Study selection

**Figure 1:**
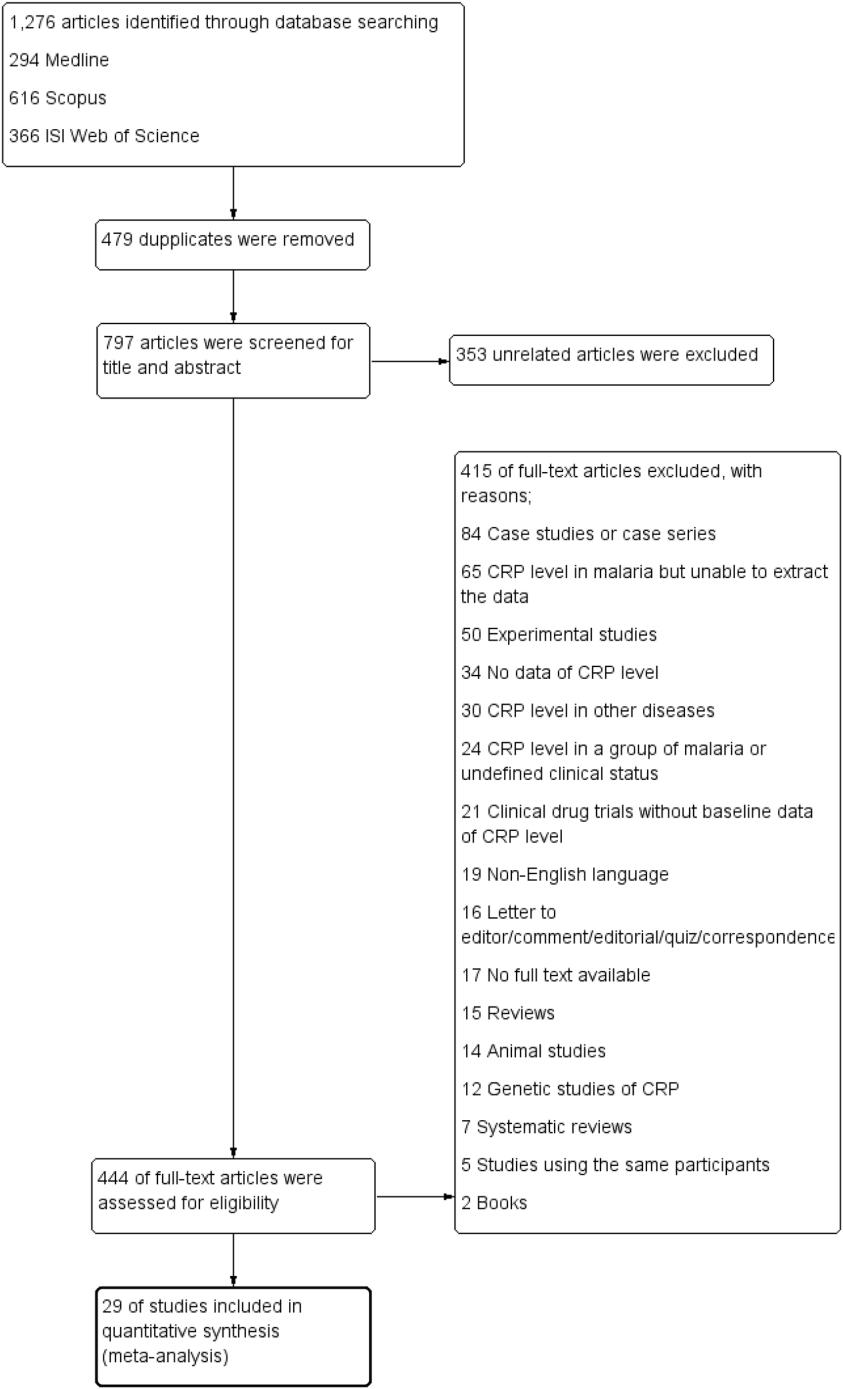
Flowchart for the study selection. Flowchart demonstrates the selection of potentially relevant studies for this study.

The potentially relevant studies were selected based on the eligibility criteria by three authors (PM, TT, and IH) and were cross-checked by another author (MK). First, the duplicate studies retrieved from the three databases were removed before study selection. Second, the relevant studies were screened for titles and abstracts. Third, if the studies met the criteria in the second step, the full text of the studies was examined. In the third step, if any study was excluded, the reasons for exclusion were provided. Finally, studies that met the inclusion and exclusion criteria were included in the quantitative synthesis. The processes of study selection are provided in the study diagram (Fig. 1).

### Data extraction

The data extraction was performed by three authors (PM, TT, and IH) and was cross-checked by another author (MK). The following data were extracted into the pilot datasheet before further analysis: the name of the first author, year of publication, study site, study design, year that the study was conducted, participants, sex, age, number of malaria cases, *Plasmodium* spp., number of controls, CRP level, and methods for CRP measurement.

### Quality of the included studies

The quality of the included studies was assessed using the Newcastle–Ottawa Scale (NOS) assessing for the quality of nonrandomized studies in meta-analyses^23^. The NOS was developed to assess the quality of nonrandomized studies based on three broad categories, namely, participants’ selection, comparability of the groups, and the ascertainment of the outcome of interest. Any study rated with at least 5 stars from a total of 7 stars indicates a high-quality study.

### Outcomes

The main outcomes were the difference in CRP level between (1) patients with severe malaria and uncomplicated malaria, (2) patients with uncomplicated malaria and asymptomatic malaria, (3) patients with uncomplicated malaria and febrile/healthy controls, and (4) patients with asymptomatic malaria and febrile/healthy controls.

### Data synthesis

In the meta-analysis, the mean and standard deviation (SD) of CRP between two groups were used to estimate the pooled standard mean difference (SMD) and 95% confidence interval (CI). The SMD and 95% CI of each outcome were estimated using the random-effects models provided in the STATA Statistical Software version 15.0 (StataCorp. 2017. Stata Statistical Software: Release 15. College Station, TX: StataCorp LLC). If the median and range of CRP were reported by the included studies, the mean and SD were calculated from the median and range as described previously^24^. Additionally, if the included studies reported separate mean and SD for the clinical symptoms of malaria including CRP in cerebral and severe anemia, the mean and SD were combined as a single “severe malaria” as described previously^25^. The heterogeneity among the included studies was assessed using the Cochrane Q and I^2^ statistics. Cochrane Q statistic with a p-value of less than 0.05 indicated a significant heterogeneity among the included studies, while I^2^ statistics of more than 50% indicated a substantial heterogeneity among the included studies. When heterogeneity was significant or substantial, the random-effects model was applied to the pooled analysis. Conversely, the fixed-effects model was applied to the pooled analysis if nonsignificant heterogeneity was demonstrated. Meta-regression analysis was performed to assess potentially important covariates that might have a substantial impact on the heterogeneity. These covariates included the mean age of participants, male percentage, continents (Asia, Africa, America, Europe), types of infection (*Plasmodium* spp.), and types of control (febrile or healthy controls). Then, subgroup analyses were conducted for these covariates. A sensitivity test was performed to assess (1) the pooled SMD after excluding studies that reported the median CRP, (2) the pooled SMD after excluding studies with six stars’ quality, and (3) the pooled SMD using a fixed-effects model compared with the random-effects model. The publication bias among the included studies was assessed by visualization of the funnel plot and Egger’s test for asymmetry. If the Egger’s test showed asymmetry, the contour-enhanced funnel plot was further assessed to determine whether the asymmetry was due to publication bias or other factors.

## Results

### Search results

The searches retrieved 1,276 articles: 294 articles from PubMed, 616 from Scopus, and 366 from ISI Web of Science. After 479 duplicated articles were removed, 797 articles were retained for review. After the title and abstract of 797 articles were screened, 353 unrelated articles were removed. Full texts of 444 articles were examined to find the potentially relevant articles, and 414 articles were excluded for the following reasons: 84 articles were case studies or case series; 65 reported CRP level in malaria but were unable to extract the data; 50 were experimental studies; 34 had no data of CRP level; 30 reported CRP level in other diseases; 24 reported CRP level in one group of malaria or undefined clinical status; 21 were clinical drug trials without baseline data of CRP level; 19 were in a non-English language; 16 were letters to the editor, comment, editorial, quiz, or correspondence; 17 had no full text available; 15 were review articles; 14 were animal studies; 12 were genetic studies of CRP; 7 were systematic reviews; 5 studies used the same participants; and 2 articles were books. Finally, 29 articles^26–54^ that reported quantitative measurement of CRP levels were included for meta-analysis (Fig. 1).

### Characteristics of the included studies

Characteristics of the included studies are shown in Table 1. All included studies reported the quantitative data of CRP in patients were published between 1989 and 2021. The included studies were conducted in four continents including Africa (14/29, 48.3%)^26,28,29,32,34,35,38–40,42,43,50,51,53^, Europe (8/29, 27.6%)^31,36,37,41,45,49,52,54^, America (3/29, 10.3%)^27,33,46^, and Asia (4/29, 13.8%)^30,44,47,48^. In Africa, studies were conducted in nine countries, namely, Gabon^42,51^, Gambia^40^, Ghana^28,38^, Kenya^29,53^, Malawi^32,35^, Nigeria^26^, Sudan^50^, Tanzania^43^, and Uganda^34^. In Europe, studies were conducted in six countries, namely, Italy^45,49^, France^31^, Germany^54^, Netherlands^52^, Sweden^36^, and United Kingdom^41^. In America, studies were conducted in Brazil^27,33,46^. In Asia, studies were conducted in three countries, namely, India^30,47^, Cambodia^48^, and Japan^44^.

**Table 1:** Characteristics of the included studies.

Parameters	Number of study (%)
**Publication years**	
1989–2000	4 (13.8%)
2001–2010	6 (20.7%)
2011–2021	19 (65.5%)
**Study locations**	
Africa	14 (48.3%)
Europe	8 (27.6%)
Asia	4 (13.8%)
America	3 (10.3%)
**Study designs**	
Prospective observational studies	14 (48.3%)
Retrospective observational studies	6 (20.7%)
Cross-sectional studies	4 (13.8%)
Cohort studies	3 (10.3%)
Case control studies	2 (6.90%)
***Plasmodium* spp.**	
*P. falciparum*	18 (62.1%)
*P. vivax*	3 (10.3%)
More than one *Plasmodium* species	8 (27.6%)
**Sign or symptoms**	
Severe malaria	12 (41.4%)
Uncomplicated malaria	18 (62.1%)
Asymptomatic malaria	12 (41.4%)
**CRP measurement**	
Turbidimetric immunoassay	8 (27.6%)
ELISA	7 (24.1%)
Fluorescence Immunoassay	2 (6.90%)
Immunoassay (not defined specific method)	2 (6.90%)
Radial immunodiffusion	1 (3.45%)
Immunologic agglutination	1 (3.45%)
Not specified	8 (27.6%)

CRP, C-reactive protein; ELISA, Enzyme-linked immunosorbent assay.

Most of the included studies (18/29, 62.1%)^26,28–32,34,35,38–40,42,43,49–53^ reported *P. falciparum* mono-infection, while three studies (10.3%)^27,33,46^ reported *P. vivax* mono-infection. Eight studies (27.6%)^36,37,41,44,45,47,48,54^ reported that malaria was caused by more than two *Plasmodium* spp. (Table S2). Among the included studies, there were 731 cases of severe malaria, 1,600 cases of uncomplicated malaria, 1,697 cases of asymptomatic malaria, and 3,658 cases of healthy/febrile controls.

### Quality of the included studies

The quality of the included studies was assessed using the NOS, which has a maximum total of 7 stars. Sixteen studies were rated 7 stars in total, while 14 studies were rated 6 stars in total as febrile patients had been used as a control group (Table S3).

### The difference in the mean CRP level between patients with severe and uncomplicated malaria

**Figure 2:**
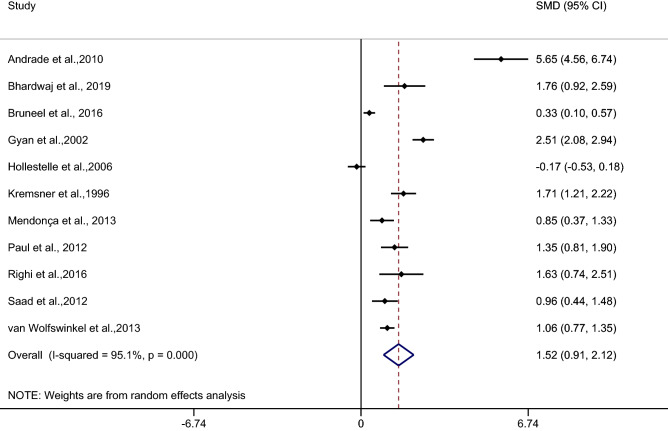
Forest plot demonstrates the difference in the mean CRP level between patients with severe and uncomplicated malaria. SMD, standard mean difference; CI, confidence interval.

The difference in the mean CRP level between patients with severe and uncomplicated malaria was estimated from 11 studies^27,30,31,38,39,42,46,47,49,50,52^. The results of the individual studies demonstrated a higher mean CRP level in patients with severe malaria than in those with uncomplicated malaria among 10 studies^27,30,31,38,42,46,47,49,50,52^. The highest SMD was reported in a study by Andrade et al.^27^, while no difference was reported in the mean CRP level in patients with severe malaria compared with those with uncomplicated malaria in a study by Hollestelle et al.^39^. The pooled analysis of 11 studies showed a higher mean CRP level in patients with severe malaria than in those with uncomplicated malaria (*p* < 0.001, SMD: 1.52, 95% CI: 0.91–2.12, I^2^: 95.1%) (Fig. 2) (Table 2).

**Table 2:** Summary of meta-analysis.

Comparisons	*p* value	SMD (95% CI)	I^2^	Number of studies for estimation
**Severe malaria vs uncomplicated malaria**	< 0.001	1.52 (0.91–2.12)	95.1	11
By continents				
Asia	< 0.001	1.47 (1.02–1.93)	0	2
Europe	0.007	0.91 (0.25–1.57)	89.7	2
Africa	0.05	1.05 (− 0.01–2.51)	96.9	4
America	0.18	3.22 (− 1.48–7.93)	98.4	2
By types of infection				
*P. falciparum*	< 0.001	1.19 (0.57–1.82)	94.06	7
*P. vivax*	0.18	3.22 (− 1.48–7.93)	98.4	2
**Uncomplicated vs asymptomatic malaria**	< 0.001	1.65 (0.67–2.62)	96.7	4
**Uncomplicated malaria vs febrile/healthy controls**	< 0.001	2.38 (1.37–3.40)	98.5	12
By types of controls				
Febrile controls	0.028	1.80 (0.19–3.4)	98.9	6
Healthy control	< 0.001	3.80 (2.78–4.83)	86	4
Febrile and healthy controls	0.172	1.32 (− 0.57–3.22)	98.7	2
By continents				
Asia	< 0.001	3.38 (3.01–3.75)	0	2
Europe	0.15	1.57 (− 0.59–3.72)	98.9	4
Africa	< 0.001	3.26 (1.52–5.01)	96.5	4
America	0.17	1.32 (− 0.57–3.22)	98.7	2
By types of infection				
*P. falciparum*	< 0.001	3.29 (1.86–4.71)	95.7	5
*P. vivax*	0.17	1.32 (− 0.57–3.22)	98.7	2
*P. falciparum*/*P. vivax*/*P. ovale*	< 0.001	3.10 (2.18–4.01)	80.4	2
**Asymptomatic malaria and febrile/healthy controls**	< 0.001	2.55 (1.6–3.5)	99.2	10
By types of controls				
Healthy control	< 0.001	3.01 (1.82–4.2)	99.3	8
Febrile and healthy controls	0.064	2.79 (1.71–3.87)	99.1	2
By continents				
Africa	0.001	3.39 (1.93–4.85)	99.2	7
America	0.76	3.22 (− 0.05–1.56)	93.7	2
By types of infection				
*P. falciparum*	< 0.001	3.39 (1.93–4.85)	99.2	7
*P. vivax*	0.064	0.76 (− 0.05–1.56)	93.7	2

Meta-regression analysis using the mean age of participants, male percentage of participants, *Plasmodium* spp., types of control, or continents as covariates demonstrated no substantial impact of the mean age of participants (*p*: 0.89, I^2^ residual: 94.5%), male percentage of participants (*p*: 0.79, I^2^ residual: 94.6%), *Plasmodium* spp. (*p*: 0.31, I^2^ residual: 95.8%), types of control (*p*: 0.59, I^2^ residual: 96.91%), or continents (*p*: 0.49, I^2^ residual: 96.07%) on the heterogeneity.

**Figure 3:**
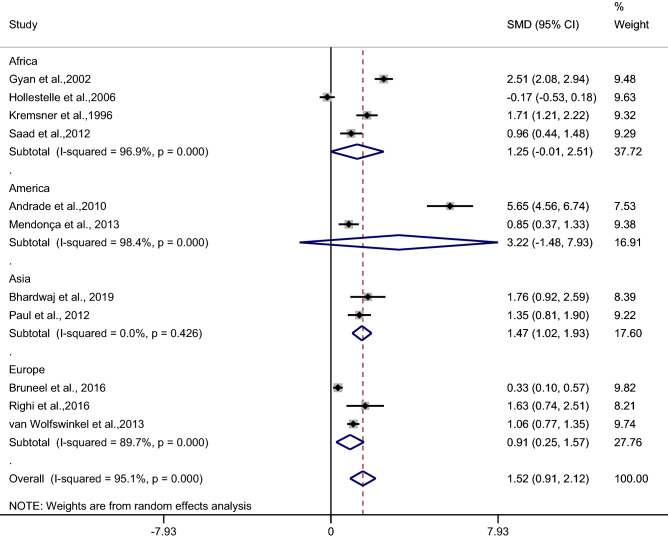
Forest plot demonstrates the difference in the mean CRP level between patients with severe and uncomplicated malaria by continents. SMD, standard mean difference; CI, confidence interval.

Subgroup analysis of continents demonstrated a higher mean CRP level in patients with severe malaria than in uncomplicated malaria patients in studies conducted in Asia (*p* < 0.001, SMD: 1.47, 95% CI: 1.02–1.93, I^2^: 0%, two studies) and in Europe (*p*: 0.007, SMD: 0.91, 95% CI: 0.25–1.57, I^2^: 89.7%, two studies). No difference was observed in the mean CRP level in patients with severe malaria compared with patients with uncomplicated malaria in studies conducted in Africa (*p*: 0.05, SMD: 1.05, 95% CI: − 0.01–2.51, I^2^: 96.9%, four studies) and America (*p*: 0.18, SMD: 3.22, 95% CI: − 1.48–7.93, I^2^: 98.4%, two studies) (Fig. 3).

**Figure 4:**
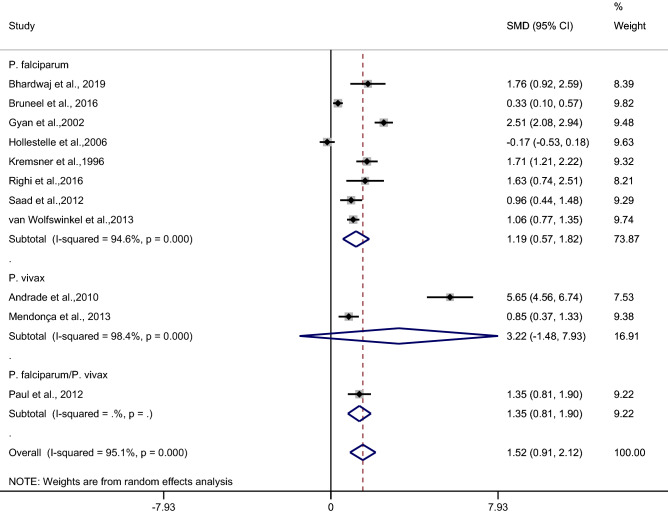
Forest plot demonstrates the difference in the mean CRP level between patients with severe and uncomplicated malaria by types of infections. SMD, standard mean difference; CI, confidence interval.

Subgroup analysis of types of infection demonstrated a higher mean CRP level in patients with severe malaria than in uncomplicated malaria patients in studies of patients with *P. falciparum* (*p* < 0.001, SMD: 1.19, 95% CI: 0.57–1.82, I^2^: 94.06%, seven studies). No difference in the mean CRP level was observed in patients with severe malaria compared with uncomplicated malaria patients in studies of patients with *P. vivax* (*p*: 0.18, SMD: 3.22, 95% CI: − 1.48–7.93, I^2^: 98.4%, two studies) (Fig. 4).

### The difference in the mean CRP level between patients with uncomplicated and asymptomatic malaria

**Figure 5:**
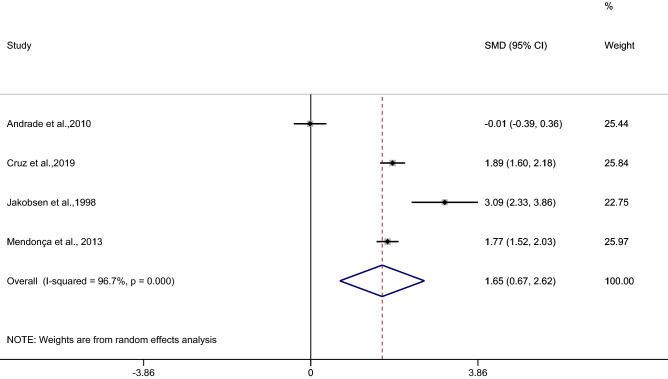
Forest plot demonstrates the difference in the mean CRP level between patients with uncomplicated and asymptomatic malaria. SMD, standard mean difference; CI, confidence interval.

The difference in the mean CRP level between patients with uncomplicated and asymptomatic malaria was estimated from four studies^27,33,40,46^. The results of the individual study demonstrated a higher mean CRP level in patients with uncomplicated malaria than in those with asymptomatic malaria among the three studies^33,40,46^, while a study by Andrade et al.^27^ showed no difference in the mean CRP level between the two groups. The highest difference in the mean CRP level was reported in a study by Jakobsen et al.^40^. The pooled analysis of the four studies showed a higher mean CRP level in patients with uncomplicated malaria than in those with asymptomatic malaria (*p* < 0.001, SMD: 1.65, 95% CI: 0.67–2.62, I^2^: 96.7%, 4 studies) (Fig. 5).

Meta-regression analysis using the mean age of participants, male percentage of participants, *Plasmodium* spp., or continents as covariates demonstrated no substantial impact of the mean age of participants (*p*: 0.48, I^2^ residual: 98.6%), male percentage of participants (*p*: 0.73, I^2^ residual: 96.3%), *Plasmodium* spp. (*p*: 0.59, I^2^ residual: 96.91%), or continents (*p*: 0.49, I^2^ residual: 96.07%) on the heterogeneity.

### The difference in the mean CRP level between patients with uncomplicated malaria and febrile/healthy controls

The difference in the mean CRP level between patients with uncomplicated malaria and febrile/healthy controls was estimated from 12 studies^26,27,30,36,37,39,40,44–46,50,54^. A meta-analysis of the difference in mean CRP level between patients with uncomplicated malaria and febrile/healthy controls were divided into three subgroups: uncomplicated malaria and febrile controls, uncomplicated malaria and healthy controls, and uncomplicated malaria and febrile/healthy controls because previous studies^27,46^ reported the mean/median CRP levels of febrile and healthy controls.

**Figure 6:**
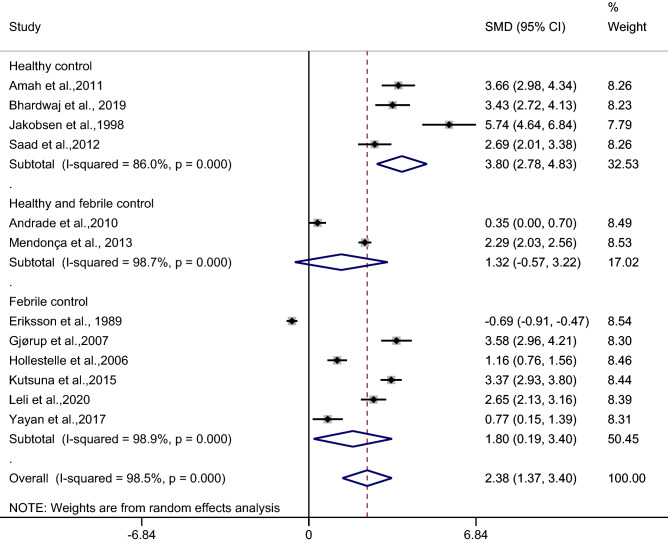
Forest plot demonstrates the difference in the mean CRP level between patients with uncomplicated malaria and healthy/febrile controls. SMD, standard mean difference; CI, confidence interval.

In the healthy control subgroup, the results of the individual study demonstrated a higher mean CRP level in patients with uncomplicated malaria than in healthy controls among the four studies^26,30,40,50^. The pooled analysis in these subgroups demonstrated a higher mean CRP level in patients with uncomplicated malaria than in healthy controls (*p* < 0.001, SMD: 3.80, 95% CI: 2.78–4.83, I^2^: 86%, 4 studies) (Fig. 6).

In the healthy and febrile control subgroup, the results of the individual study demonstrated a higher mean CRP level in patients with uncomplicated malaria than in febrile/healthy controls in a study by Mendonça et al.^46^, while no difference was shown in a study by Andrade et al.^27^. The pooled analysis in these subgroups demonstrated no difference in the mean CRP level in patients with uncomplicated malaria than in febrile/healthy controls (*p*: 0.172, SMD: 1.32, 95% CI: − 0.57–3.22, I^2^: 98.7%, 2 studies).

In the febrile subgroup, the results of the individual study demonstrated a higher mean CRP level in patients with uncomplicated malaria than in febrile controls among five studies^37,39,44,45,54^, while a lower mean CRP level in patients with uncomplicated malaria than in febrile controls was demonstrated in a study by Eriksson et al.^36^. The pooled analysis in these subgroups demonstrated a higher mean CRP level in patients with uncomplicated malaria than in febrile controls (*p*: 0.028, SMD: 1.80, 95% CI: 0.19–3.4, I^2^: 98.9%, 6 studies).

Overall, the pooled analysis of 12 studies demonstrated a higher mean CRP level in uncomplicated malaria than in febrile/healthy controls (*p* < 0.001, SMD: 2.38, 95% CI: 1.37–3.40, I^2^: 98.5%, 12 studies).

Meta-regression analysis using the mean age of participants, male percentage of participants, *Plasmodium* spp., types of control, or continents as covariates demonstrated no substantial impact of the mean age of participants (*p*: 0.072, I^2^ residual: 98.75%), male percentage of participants (*p*: 0.53, I^2^ residual: 98.53%), *Plasmodium* spp. (*p*: 0.23, I2 residual: 96.56%), types of control (*p*: 0.12, I^2^ residual: 98.31%), or continents (*p*: 0.39, I^2^ residual: 98.11%) on the heterogeneity.

**Figure 7:**
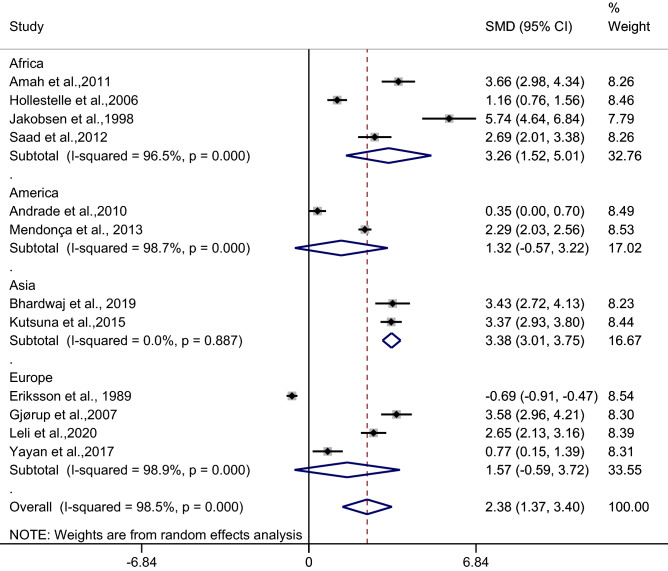
Forest plot demonstrates the difference in the mean CRP level between patients with uncomplicated malaria and healthy/febrile controls by continents. SMD, standard mean difference; CI, confidence interval.

Subgroup analysis of continents demonstrated a higher mean CRP level in patients with uncomplicated malaria than in febrile/healthy controls in studies conducted in Africa (*p* < 0.001, SMD: 3.26, 95% CI: 1.52–5.01, I^2^: 96.5%, four studies) and Asia (*p* < 0.001, SMD: 3.38, 95% CI: 3.01–3.75, I^2^: 0%, two studies). No difference in the mean CRP level was observed in patients with uncomplicated malaria and febrile/healthy controls in studies conducted in Europe (*p*: 0.15, SMD: 1.57, 95% CI: − 0.59–3.72, I^2^: 98.9%, four studies) and America (*p*: 0.17, SMD: 1.32, 95% CI: − 0.57–3.22, I^2^: 98.7%, two studies) (Fig. 7).

**Figure 8:**
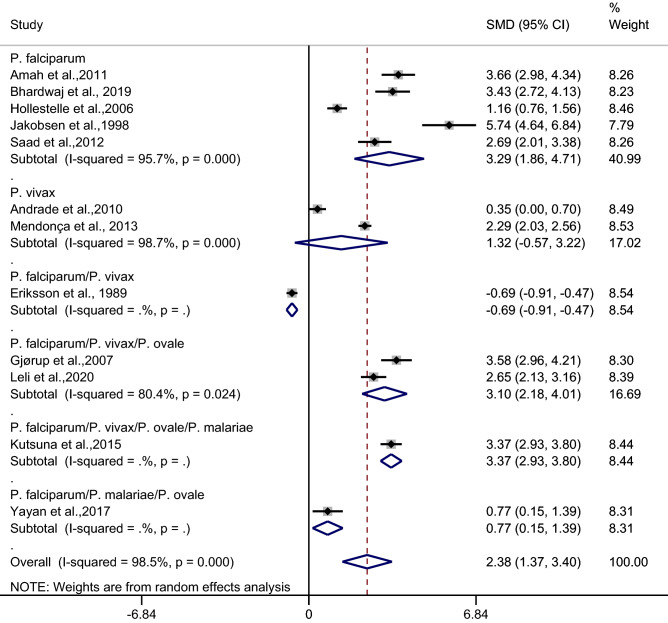
Forest plot demonstrates the difference in the mean CRP level between patients with uncomplicated malaria and healthy/febrile controls by types of infections. SMD, standard mean difference; CI, confidence interval.

Subgroup analysis of types of infection demonstrated a higher mean CRP level in patients with uncomplicated malaria than in febrile/healthy controls in studies of patients with *P. falciparum* (*p* < 0.001, SMD: 3.29, 95% CI: 1.86–4.71, I^2^: 95.7%, five studies) and studies of patients with *P. falciparum*/*P. vivax*/*P. ovale* (*p* < 0.001, SMD: 3.10, 95% CI: 2.18–4.01, I^2^: 80.4%, two studies). No difference in the mean CRP level was observed in patients with uncomplicated malaria and febrile/healthy controls in studies of *P. vivax* (*p*: 0.17, SMD: 1.32, 95% CI: − 0.57–3.22, I^2^: 98.7%, two studies) (Fig. 8).

### The difference in the mean CRP level between asymptomatic malaria patients and febrile/healthy controls

**Figure 9:**
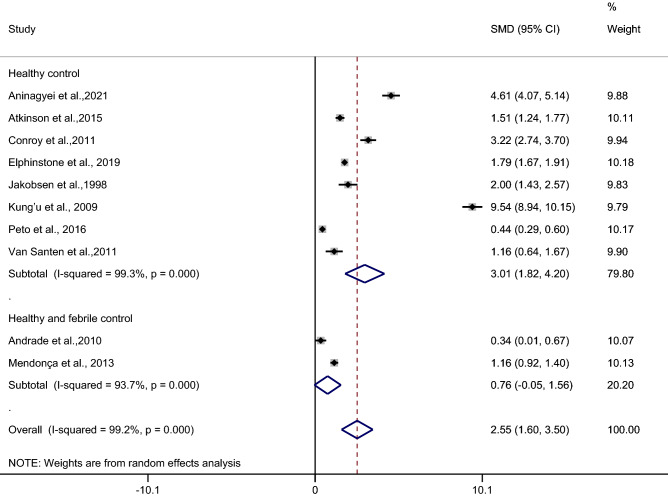
Forest plot demonstrates the difference in the mean CRP level between patients with asymptomatic malaria and healthy/febrile controls. SMD, standard mean difference; CI, confidence interval.

The difference in the mean CRP level between asymptomatic malaria patients and febrile/healthy controls was estimated using 10 studies^27–29,32,35,40,43,46,48,51^. Results of the individual study demonstrated a higher mean CRP level in asymptomatic malaria patients than in febrile/healthy controls in all 10 studies^27–29,32,35,40,43,46,48,51^. The highest difference in the mean CRP level between asymptomatic malaria patients and healthy controls was demonstrated in a study by Kung’u et al.^43^. Subgroup analysis showed a higher mean CRP level in asymptomatic malaria patients than in healthy controls (*p* < 0.001, SMD: 3.01, 95% CI: 1.82–4.2, I^2^: 99.3%, 8 studies), but no difference was observed in the mean CRP level between asymptomatic malaria patients and febrile/healthy controls (*p*: 0.064, SMD: 2.79, 95% CI: 1.71–3.87, I^2^: 99.1%, 2 studies). Overall, the pooled analysis demonstrated a higher mean CRP level in asymptomatic malaria patients than in healthy controls (*p* < 0.001, SMD: 2.55, 95% CI: 1.6–3.5, I^2^: 99.2%, 10 studies) (Fig. 9).

Meta-regression analysis using the mean age of participants, male percentage of participants, *Plasmodium* spp., types of control, or continents as covariates demonstrated no substantial impact of the mean age of participants (*p*: 0.53, I^2^ residual: 99.3%), male percentage of participants (*p*: 0.65, I^2^ residual: 99.3%), *Plasmodium* spp. (*p*: 0.39, I^2^ residual: 99.08%), types of control (*p*: 0.32, I^2^ residual: 99.25%), or continents (*p*: 0.39, I^2^ residual: 99.08%) on the heterogeneity.

**Figure 10:**
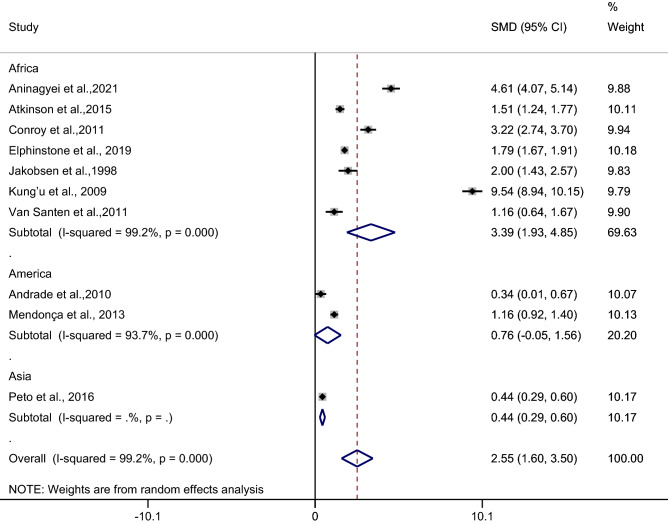
Forest plot demonstrates the difference in the mean CRP level between patients with asymptomatic malaria and healthy/febrile controls by continents. SMD, standard mean difference; CI, confidence interval.

Subgroup analysis of continents demonstrated a higher mean CRP level in patients with asymptomatic malaria than in febrile/healthy controls in studies conducted in Africa (*p* < 0.001, SMD: 3.39, 95% CI: 1.93–4.85, I^2^: 99.2%, seven studies) whereas no difference in the mean CRP level was observed in patients with asymptomatic malaria compared with febrile/healthy controls in studies conducted in America (*p*: 0.76, SMD: 3.22, 95% CI: − 0.05–1.56, I^2^: 93.7%, two studies) (Fig. 10).

**Figure 11:**
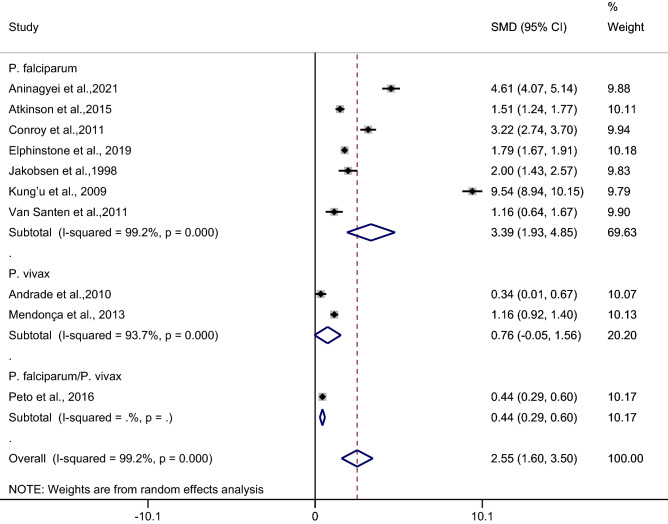
Forest plot demonstrates the difference in the mean CRP level between patients with asymptomatic malaria and healthy/febrile controls by types of infections. SMD, standard mean difference; CI, confidence interval.

Subgroup analysis of types of infection demonstrated a higher mean CRP level in patients with asymptomatic malaria than in febrile/healthy controls in studies of patients with *P. falciparum* (*p* < 0.001, SMD: 3.39, 95% CI: 1.93–4.85, I^2^: 99.2%, seven studies). No difference in the mean CRP level was observed in patients with asymptomatic malaria compared with febrile/healthy controls in studies of *P. vivax* (*p*: 0.064, SMD: 0.76, 95% CI: − 0.05–1.56, I^2^: 93.7%, two studies) (Fig. 11).

### Sensitivity analysis

1. Studies reporting the median CRP were excluded.

Differences in CRP levels between patients with severe and uncomplicated malaria were estimated after studies reporting the mean CRP were excluded. Results demonstrated a higher mean CRP level in patients with severe malaria than in those with uncomplicated malaria (*p*: 0.006, SMD: 1.21, 95% CI: 0.35–2.06, I^2^: 96%, six studies) (Supplementary Fig. 1).

2. Studies with six stars’ quality were excluded.

Differences in CRP levels between patients with severe and uncomplicated malaria were estimated after studies with six stars’ quality were excluded. Results demonstrated a higher mean CRP level in patients with severe malaria than in those with uncomplicated malaria (*p*: 0.001, SMD: 1.72, 95% CI: 0.41–3.04, I^2^: 96.3%, five studies) (Supplementary Fig. 2).

3. Fixed-effects model and random-effects model.

A pooled analysis using the fixed-effects model demonstrated a higher mean CRP level in patients with severe malaria than in those with uncomplicated malaria (*p* < 0.001, SMD: 0.93, 95% CI: 0.81–1.06, I^2^: 95.1%) (Supplementary Fig. 3). A pooled analysis using the fixed-effects model demonstrated a higher mean CRP level in patients with uncomplicated malaria than in those with asymptomatic malaria (*p* < 0.001, SMD: 1.52, 95% CI: 1.36–1.69, I^2^: 96.7%) (Supplementary Fig. 4). A pooled analysis using the fixed-effects model demonstrated a higher mean CRP level in patients with uncomplicated malaria than in healthy/febrile controls (*p* < 0.001, SMD: 1.49, 95% CI: 1.42–1.57, I^2^: 99.2%) (Supplementary Fig. 5).

### Publication bias

Publication bias was assessed by visualization of a funnel plot for asymmetry. The funnel plot was plotted using the effect size (SMD) and the standard error of the effect size.

1. Severe and uncomplicated malaria.

**Figure 12:**
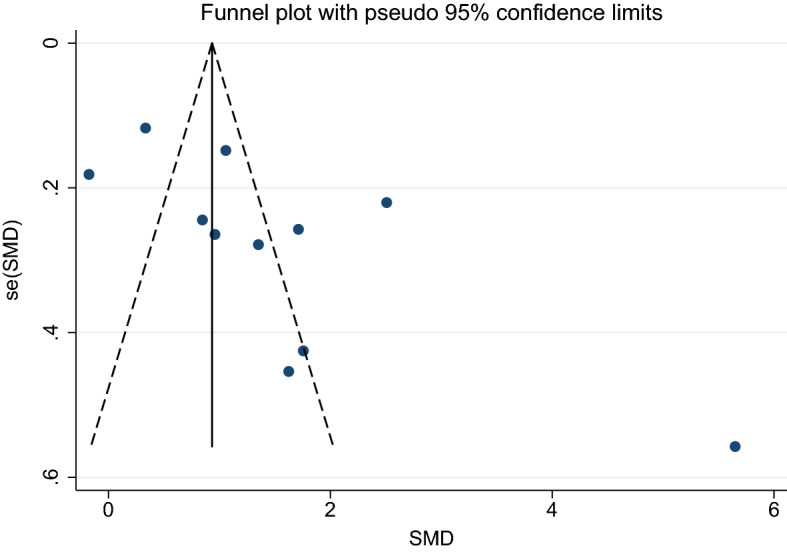
Funnel plot demonstrates the publication bias among 11 studies comparing the mean CRP level between patients with severe and uncomplicated malaria. SMD, standard mean difference; se, standard error.

Using the effect size from 11 studies, the funnel plot demonstrated an asymmetrical distribution indicating publication bias among the included studies. Egger’s test demonstrated significant small-study effects (*p*: 0.026) among the 11 studies, indicating publication bias (Fig. 7). A trim-and-fill analysis was conducted. Results showed no difference in the mean CRP level in patients with severe malaria and those with uncomplicated malaria as estimated by the random-effects model (*p*: 0.05, SMD: 0.64, 95% CI: − 0.003–1.28, 16 studies). However, there was a higher mean CRP level in patients with severe malaria than in those with uncomplicated malaria as estimated by the fixed-effects model (*p* < 0.001, SMD: 0.59, 95% CI: 0.47–0.70, 16 studies) (Fig. 12).

2. Uncomplicated malaria and asymptomatic malaria.

**Figure 13:**
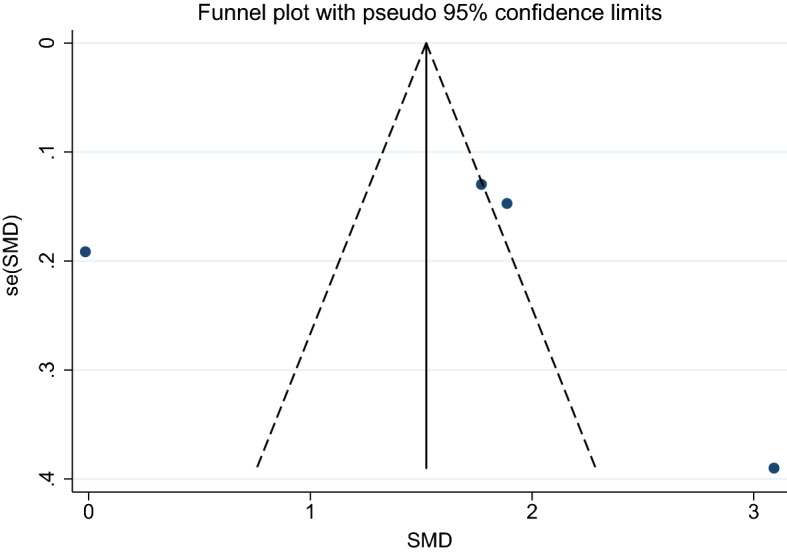
Funnel plot demonstrates the publication bias among 11 studies comparing the mean CRP level between patients with uncomplicated and asymptomatic malaria. SMD, standard mean difference; se, standard error.

Using the effect size from four studies, the funnel plot demonstrated an asymmetrical distribution indicating publication bias among the included studies (Fig. 13). Egger’s test demonstrated no significant small-study effects (*p*: 0.94) among the four studies, indicating no publication bias. Therefore, a trim-and-fill analysis was not conducted.

3. Uncomplicated malaria and febrile/healthy controls.

**Figure 14:**
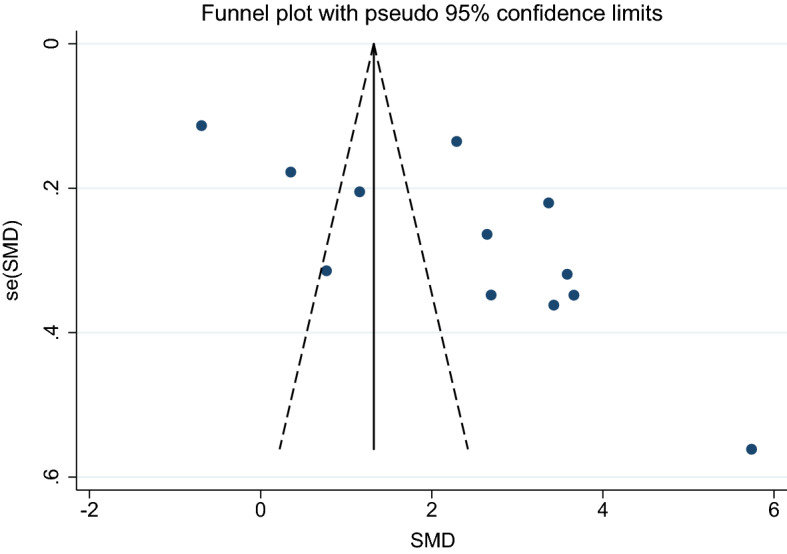
Funnel plot demonstrates the publication bias among 11 studies comparing the mean CRP level between patients with uncomplicated and healthy/febrile controls. SMD, standard mean difference; se, standard error.

Using the effect size from 12 studies, the funnel plot demonstrated an asymmetrical distribution indicating publication bias among the included studies. Egger’s test demonstrated significant small-study effects (*p*: 0.012) among the 12 studies (Fig. 14), indicating publication bias. A trim-and-fill analysis was conducted. Results showed a higher mean CRP level in patients with uncomplicated malaria than in those with febrile/healthy controls as estimated by the random-effects model (*p*: 0.04, SMD: 1.02, 95% CI: − 0.031–2.00, 17 studies) and by the fixed-effects model (*p* < 0.001, SMD: 0.81, 95% CI: 0.7–0.92, 17 studies).

4. Asymptomatic malaria and febrile/healthy controls.

**Figure 15:**
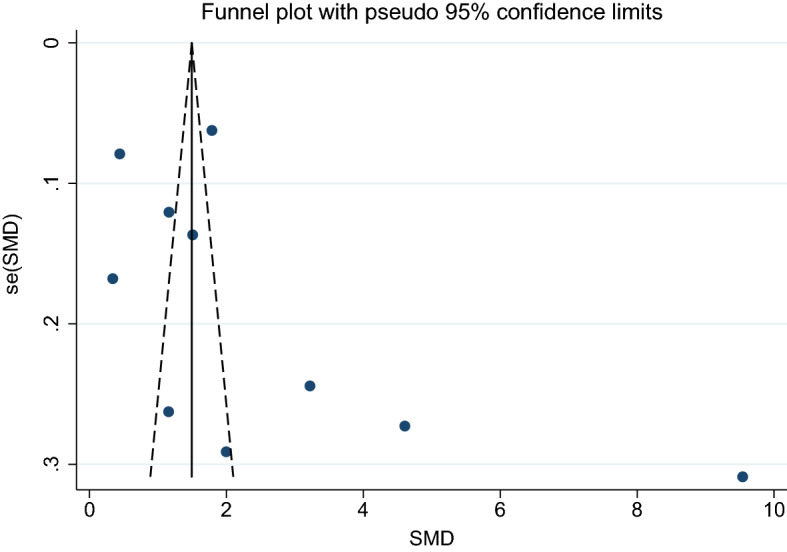
Funnel plot demonstrates the publication bias among 11 studies comparing the mean CRP level between patients with asymptomatic malaria and healthy/febrile controls. SMD, standard mean difference; se, standard error.

Using the effect size from 10 studies, the funnel plot demonstrated an asymmetrical distribution indicating publication bias among the included studies. Egger’s test demonstrated no significant small-study effects (*p*: 0.152) among the 10 studies, indicating no publication bias (Fig. 15). A trim-and-fill analysis was not conducted.

## Discussion

In this study, our meta-analysis demonstrated that CRP levels were significantly higher in patients with severe malaria than in those with uncomplicated malaria, in patients with uncomplicated malaria than in those with asymptomatic malaria, in patients with uncomplicated malaria than in febrile or healthy controls, and in patients with asymptomatic malaria than in febrile or healthy controls. These results suggest CRP might be used as an early biomarker for malaria infection and the monitoring of malaria severity.

When using CRP level as a marker of malaria, a previous study showed that a cut-off value for CRP level of 10.8 mg/L could discriminate malaria from healthy controls^55^. Nevertheless, another study conducted in Southern Uganda demonstrated that about half of patients (58%) with malaria had elevated serum CRP levels greater than 50 µg/L, whereas 62% of patients without malaria had elevated CRP levels above 50 µg/L, indicating a poor cut-off CRP level for discriminating malaria from non-malaria^56^. A previous study conducted in Ghana reported low positive predictive values of elevated CRP for malaria (32%) and suggested that CRP was not useful for predicting parasitemia and malaria^57^. Another study demonstrated an increased CRP level during malaria infection but it had a low specificity to differentiate malaria from septicemia^57^. Furthermore, increased CRP levels were found in bacterial infections^14,57,58^. Although increased CRP had a low positive predictive value for malaria, a previous study demonstrated the high negative predictive value of elevated CRP for malaria (97.0%), indicating the likelihood of no malarial parasites in the blood of patients with normal CRP level, allowing malaria to be ruled out in febrile patients^57^. Therefore, the potential usefulness of CRP includes ruling out other febrile illnesses in areas where malaria is endemic such as in sub-Saharan Africa. However, further studies are required to explain how inflammatory responses compared with non-malarial inflammatory responses during malaria lead to a distinct pattern. This information might help clinicians make decisions about the diagnosis of febrile conditions in highly malaria-endemic areas and to treat patients accordingly.

A previous study conducted in Asian countries including Cambodia, Laos, and Thailand suggested elevated CRP might be used to discriminate malaria from viral infections although it could not discriminate malaria from bacterial infections^16^. Another study suggested that CRP in combination with hematological parameters including thrombocytopenia and/or leukopenia could differentiate between malaria, dengue, and enteric fever^59^. In addition, a CRP level greater than 5 mg/L could discriminate malaria from dengue infection with a sensitivity of 95% but with a poor specificity of 35%^59^. Another study suggested that a low CRP level suggested dengue fever whereas a lower mean platelet count combined with an elevated CRP level was more indicative of malaria infection in areas where both malaria and dengue were endemic^60^.

When using CRP level as a marker of malaria severity, a previous study showed that a cut-off value for CRP level of 18.5 mg/L discriminated severe malaria from uncomplicated malaria with 71.4% sensitivity and 68.7% specificity^55^. Although a CRP level less than 20 mg/L was suggested to be a strong indicator of uncomplicated malaria^61^, increased CRP levels correlated strongly with malarial parasite density, suggesting it also correlated with malaria severity^62^. In addition, increased CRP levels were found in patients with multiple complications compared with those with a single complication, and a higher CRP level was observed in patients who died compared to those who survived^47^. Furthermore, an increased CRP level was associated with a low hemoglobin concentration and a longer hospital stay, indicating a poor outcome for patients with malaria^63^. A combination of increased CRP level and other routine laboratory parameters might improve the ability of CRP to differentiate between severe and uncomplicated malaria. For example, a combination of elevated CRP with an erythrocyte sedimentation rate (ESR) greater than 34.5 mm in the first hour of diagnosis helped identify patients with uncomplicated malaria who might subsequently develop severe malaria^64^. The results of the meta-analysis supported that the elevated levels of CRP may help in the prognosis of disease severity among patients infected with malaria. However, a lower CRP concentration in patients with severe and fatal malaria than in those with uncomplicated malaria had been observed, suggesting the inability to control the inflammatory response to infection; this may be particularly important for protection against cerebral malaria^65^. The role of CRP in malaria that is linked to the severity of the disease is correlated with nitrox oxide (NO), which is a toxic substance against *P. falciparum*^66^. Additionally, increased NO could activate neurons and damage erythrocytes, which might contribute to severe anemia or cerebral malaria in patients with severe malaria^67^.

A previous study reported that, when using CRP level as a marker of asymptomatic malaria, prolonged exposure to *Plasmodium* infections among asymptomatic malaria patients resulted in a chronic inflammatory response although the median CRP value in asymptomatic malaria patients did not differ from that in the healthy population^63^. In addition, CRP levels in asymptomatic malaria patients did not vary with high density parasitemia (more than 10,000 parasites/µL) and remained less than 3.5 µg/mL compared with uncomplicated malaria patients in whom the median CRP concentration was 116.4 µg/mL, indicating that high parasitemia alone did not stimulate an acute-phase response^63^. These results indicated that CRP could be a marker of malaria infection in those participants who live in communities in endemic areas without signs or symptoms of malaria.

Besides the usefulness of CRP as an early marker for malarial infection and severity, it could be used as a prognostic marker for the efficacy of malaria treatment as CRP level was reported to be decreased under malaria treatment^68,69^. Therefore, CRP measurement may be useful for the physician to follow-up on the efficacy of treatment of malaria. However, the use of CRP as a marker of malaria infection needs to be interpreted in combination with other parameters, including procalcitonin^69^, haptoglobin^70^, serum hepcidin^71^, blood transaminases^66^, and blood count parameters^3,4,60,72,73^, to help increase the sensitivity of this marker. A high CRP level with other routine laboratory parameters could help differentiate patients with uncomplicated malaria from those with asymptomatic malaria.

In Africa, where malaria is endemic, CRP was reported to aid the diagnosis of neonatal sepsis^74–76^. In addition, a combination of CRP and procalcitonin improved the accuracy of the diagnosis of neonatal sepsis^77,78^. A study of 624 apparently healthy volunteers in Ghana reported low circulating CRP levels in the healthy Ghanaian population, indicating an adverse environmental condition in a malaria-endemic area^79^. Therefore, interpreting the differences in CRP levels among different ethnic groups living in the same country needs to be considered. Differences in CRP, particularly hs-CRP, which can detect a low amount of CRP (1–10 mg/L)^80^, were well-described in a meta-analysis involving 18,585 participants of African ancestry that reported higher hs-CRP levels in black residents in the United States compared with Hispanics, South Asians, Caucasians, and East Asians^81^. Nevertheless, in Africa hs-CRP appears to be a good marker for the early detection of malaria in asymptomatic individuals. Further studies are required to investigate the performance of hs-CRP to discriminate individuals with asymptomatic malaria from healthy individuals in those areas.

This study has several limitations. First, a high heterogeneity among the included studies was observed. Although meta-regression and subgroup analyses of age, male percentage, continents, types of infection, or type of controls were performed, the heterogeneity was high, although the source of heterogeneity could not be explored; therefore, the pooled analysis needs to be carefully interpreted. Second, the number of studies included in each analysis was limited because some relevant studies were excluded due to incomplete data for CRP level or clinical status of patients with malaria presented in the literature. Third, there was publication bias among the included studies that analyzed differences in the mean CRP level between patients with severe malaria and uncomplicated malaria, as well as between uncomplicated malaria and febrile/healthy controls; therefore, the results should be interpreted with caution. The present study indicates that CRP measurement in a routine laboratory could help clinicians in highly malaria-endemic areas to recognize malaria infection and determine its severity in patients. The physician can then make decisions on treatment accordingly.

## Conclusion

This study demonstrated the possibility of CRP as a biomarker of malaria infection and severity. Using CRP in combination with other routine laboratory parameters could serve as a biomarker for the early detection and monitoring of malaria severity. Further studies with large sample sizes are needed to determine the performance of CRP to help clinicians in highly malaria-endemic areas make appropriate decisions on malaria treatment.

## Supplementary Information

Supplementary Information 1.

Supplementary Figure S1.

Supplementary Figure S2.

Supplementary Figure S3.

Supplementary Figure S4.

Supplementary Figure S5.

Supplementary Table S1.

Supplementary Table S2.

Supplementary Table S3.

## Data Availability

All data relating to this study in this manuscript are available.
