# Supplementary figures and images for: C-reactive protein as an early biomarker for malaria infection and monitoring of malaria severity: a meta-analysis
Source: Sci Rep. 2021 Nov 11;11:22033. doi: 10.1038/s41598-021-01556-0 (PMC8585865; doi:10.1038/s41598-021-01556-0)

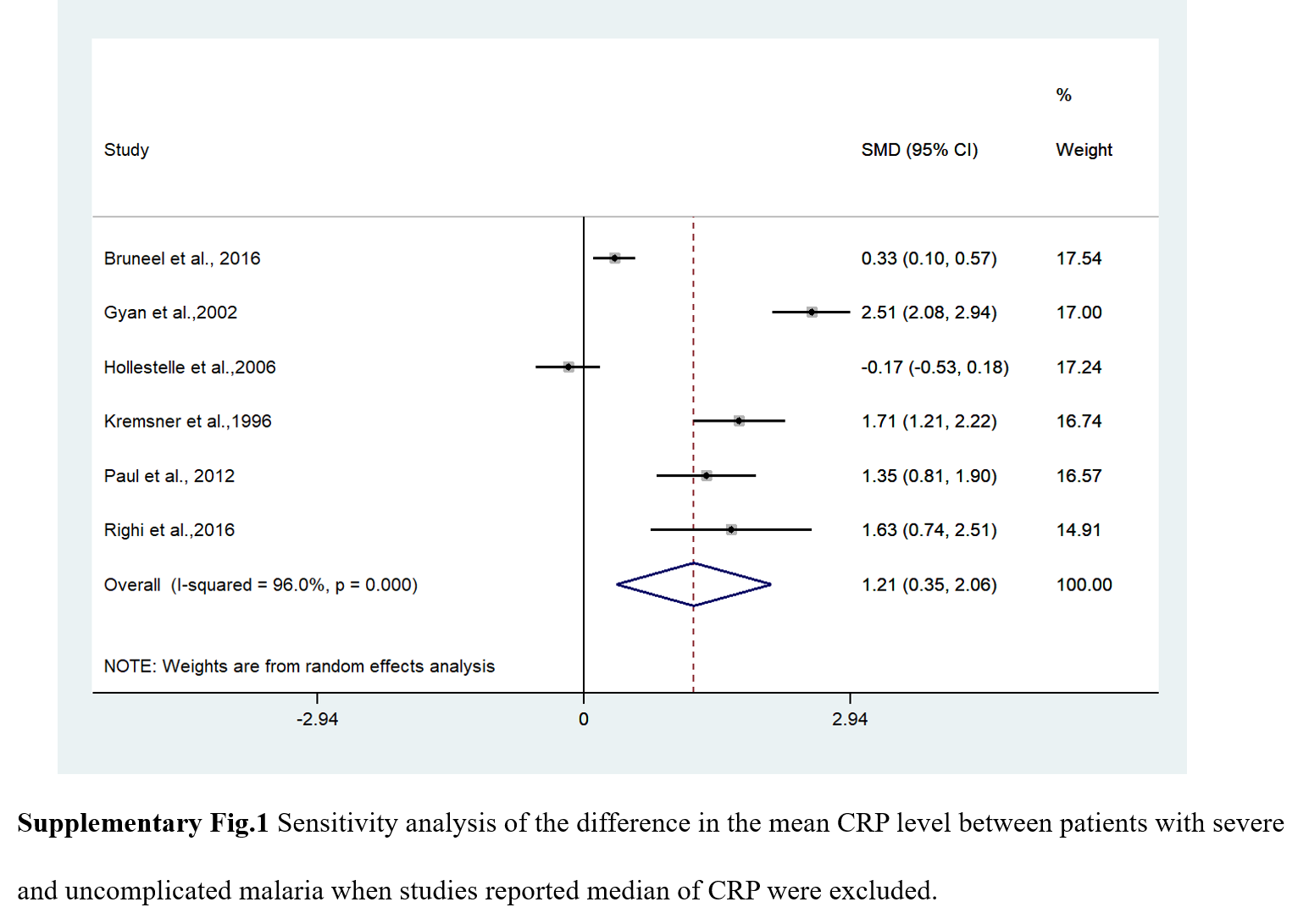

Supplement: Supplementary file 2 — Supplementary Figure S1. [file 41598_2021_1556_MOESM2_ESM.png]

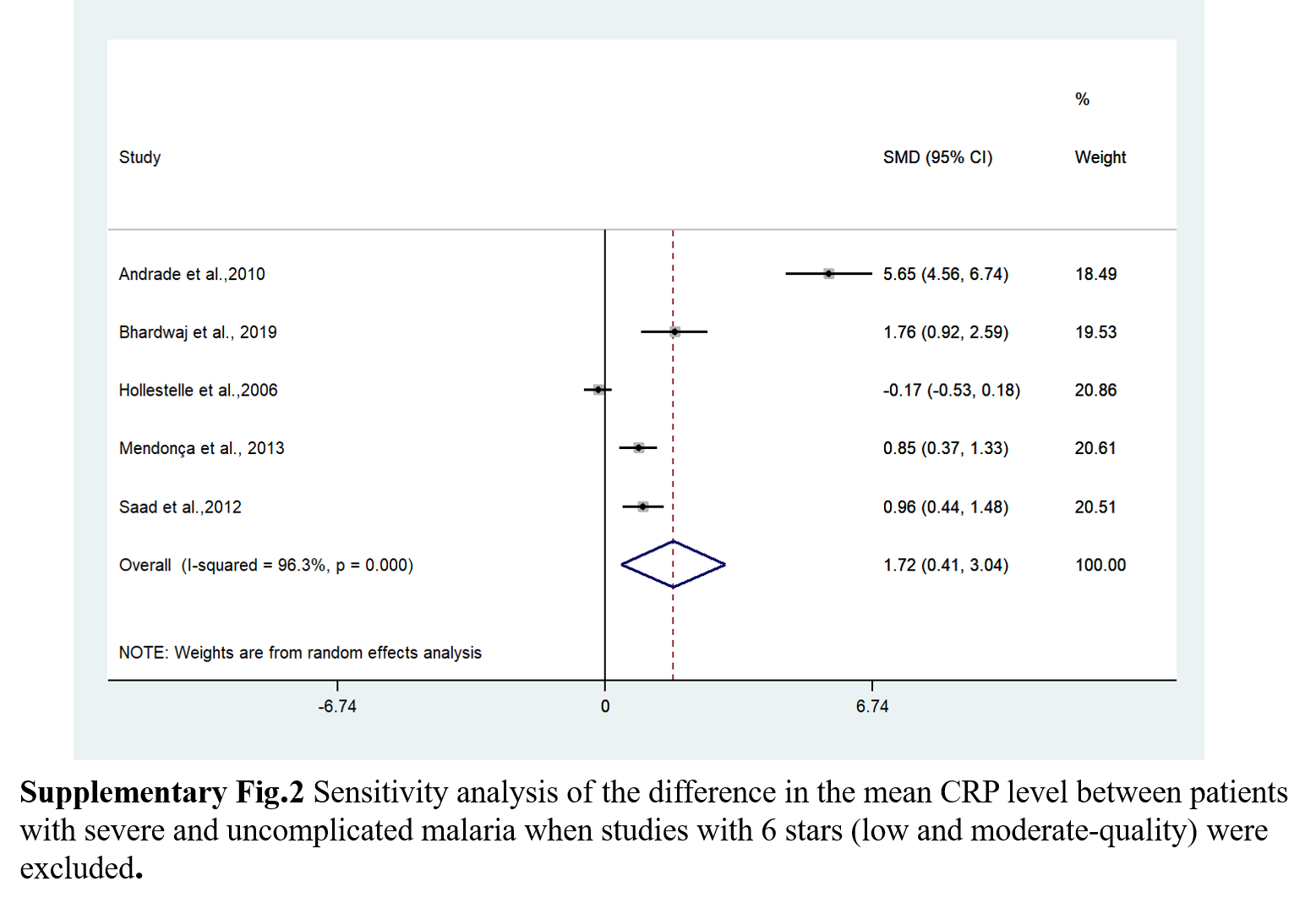

Supplement: Supplementary file 3 — Supplementary Figure S2. [file 41598_2021_1556_MOESM3_ESM.png]

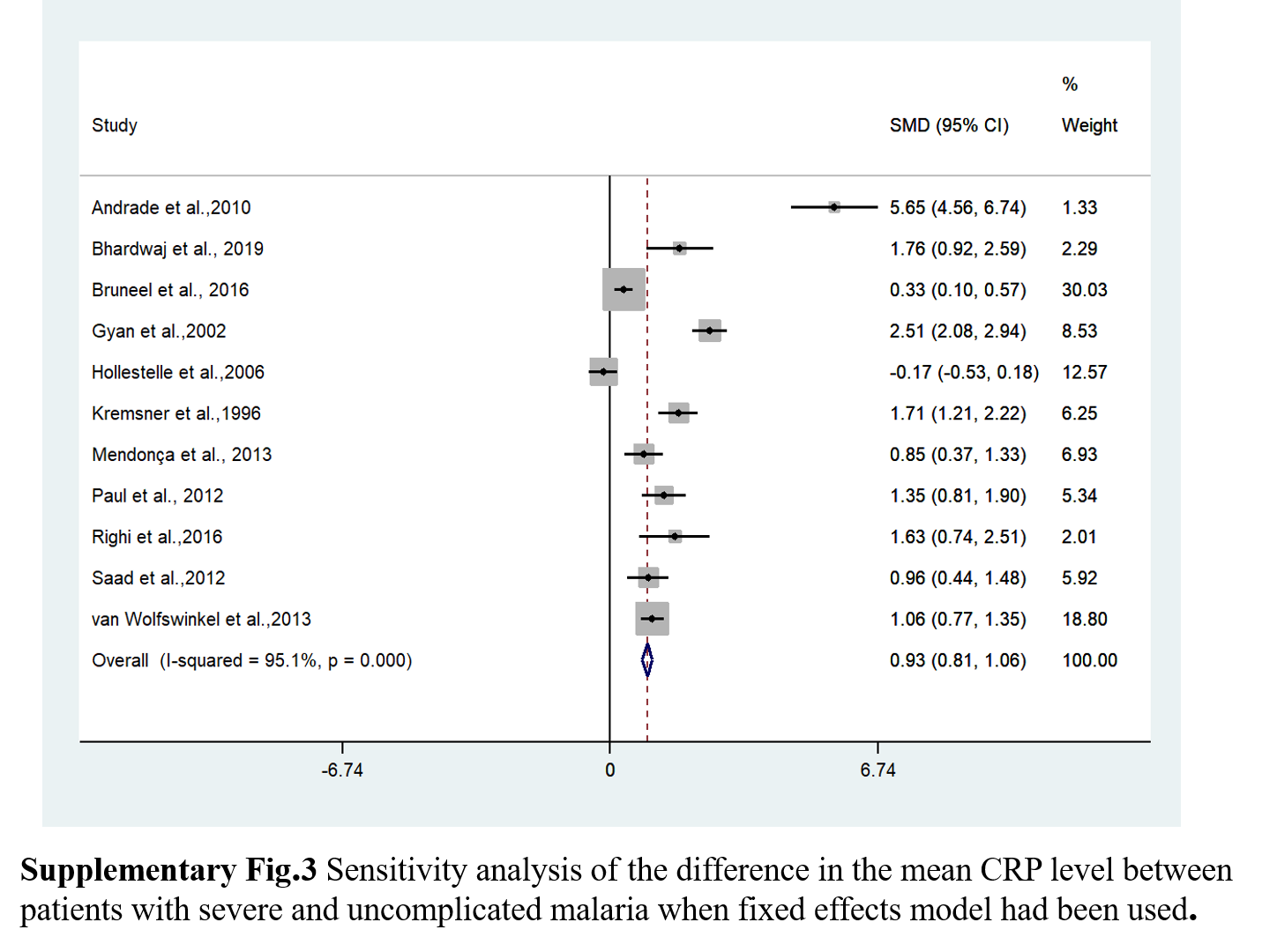

Supplement: Supplementary file 4 — Supplementary Figure S3. [file 41598_2021_1556_MOESM4_ESM.png]

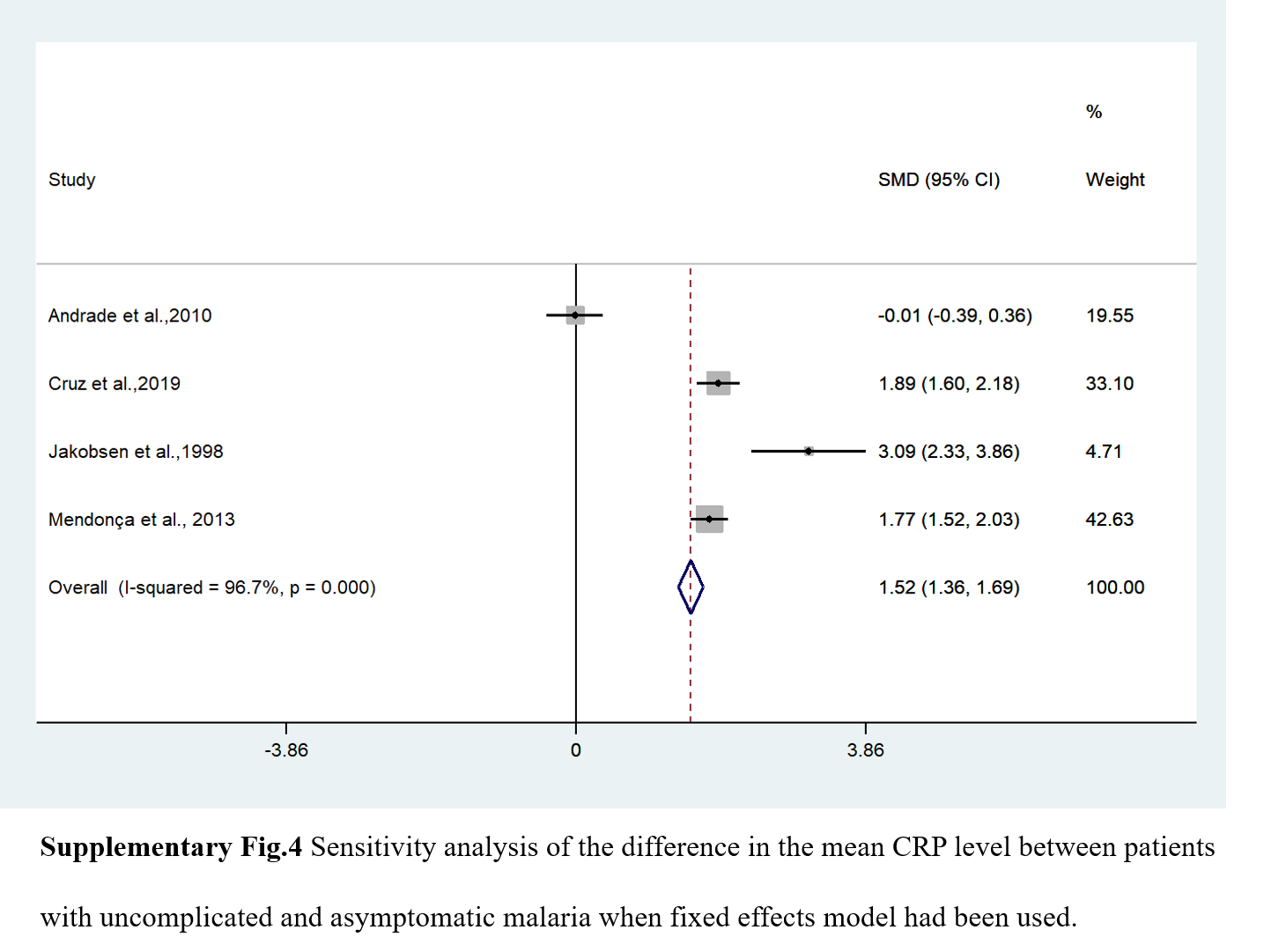

Supplement: Supplementary file 5 — Supplementary Figure S4. [file 41598_2021_1556_MOESM5_ESM.png]

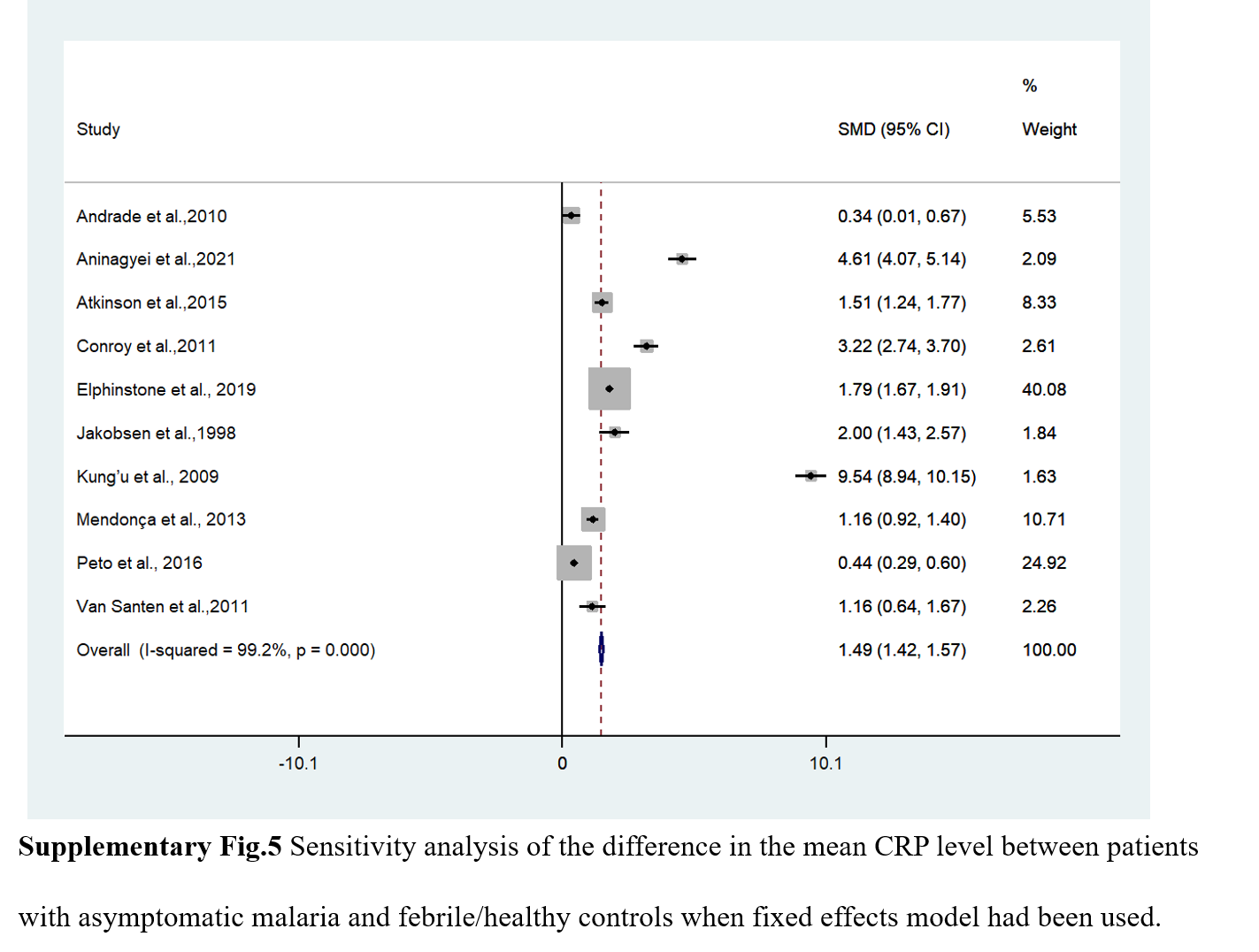

Supplement: Supplementary file 6 — Supplementary Figure S5. [file 41598_2021_1556_MOESM6_ESM.png]
