# Supplementary material for: C-reactive protein as an early biomarker for malaria infection and monitoring of malaria severity: a meta-analysis
Source: Sci Rep. 2021 Nov 11;11:22033. doi: 10.1038/s41598-021-01556-0 (PMC8585865; doi:10.1038/s41598-021-01556-0)
Supplement: Supplementary file 7 — Supplementary Table S1. [file 41598_2021_1556_MOESM7_ESM.docx]

**C-reactive protein as an early biomarker for malaria infection and monitoring of malaria severity: A meta-analysis**

Polrat Wilairatana^1^, Praphassorn Mahannop^2^, Thanita Tussato^2^, I-mee Hayeedoloh^2^, Rachasak Boonhok, Wiyada Kwanhian Klangbud, Kwuntida Uthaisar Kotepui^2^, Manas Kotepui^2^*

^1^Department of Clinical Tropical Medicine, Faculty of Tropical Medicine, Mahidol University, Bangkok, Thailand

^2^Medical Technology, School of Allied Health Sciences, Walailak University, Tha Sala, Nakhon Si Thammarat, Thailand

Authors’ Email Addresses:

**^*^Corresponding Author**: Manas Kotepui; manas.ko@wu.ac.th

Polrat Wilairatana; polrat.wil@mahidol.ac.th

Praphassorn Mahannop; praphassornmahannop367@gmail.com

Thanita Tussato; thanita.tu@mail.wu.ac.th

I-mee Hayeedoloh; imee.ha@mail.wu.ac.th

Rachasak Boonhok; rachasak.bo@wu.ac.th

Wiyada Kwanhian Klangbud; kwiyada@wu.ac.th

Wanida Mala; wanida.ma@wu.ac.th

Kwuntida Uthaisar Kotepui; kwuntida.ut@wu.ac.th

**Table S1. Search terms**

| **Databases** | **Search terms** | **Search date** |
| --- | --- | --- |
| MEDLINE (PubMed) | ("C reactive protein" OR "C-reactive protein" OR CRP OR hsCRP OR "hs-CRP") AND (malaria OR Plasmodium)  Search option: All fields | 26 January 2021 |
| Scopus | ("C reactive protein" OR "C-reactive protein" OR CRP OR hsCRP OR "hs-CRP") AND (malaria OR Plasmodium)  Search option: title, abstract, keywords | 26 January 2021 |
| ISI Web of Science | ("C reactive protein" OR "C-reactive protein" OR CRP OR hsCRP OR "hs-CRP") AND (malaria OR Plasmodium)  Search option: All fields | 26 January 2021 |
