# Supplementary material for: C-reactive protein as an early biomarker for malaria infection and monitoring of malaria severity: a meta-analysis
Source: Sci Rep. 2021 Nov 11;11:22033. doi: 10.1038/s41598-021-01556-0 (PMC8585865; doi:10.1038/s41598-021-01556-0)
Supplement: Supplementary file 8 — Supplementary Table S2. [file 41598_2021_1556_MOESM8_ESM.docx]

**C-reactive protein as an early biomarker for malaria infection and monitoring of malaria severity: A meta-analysis**

Polrat Wilairatana^1^, Praphassorn Mahannop^2^, Thanita Tussato^2^, I-mee Hayeedoloh^2^, Rachasak Boonhok^2^, Wiyada Kwanhian Klangbud^2^, Wanida Mala^2^, Kwuntida Uthaisar Kotepui^2^, Manas Kotepui^2^*

^1^Department of Clinical Tropical Medicine, Faculty of Tropical Medicine, Mahidol University, Bangkok, Thailand

^2^Medical Technology, School of Allied Health Sciences, Walailak University, Tha Sala, Nakhon Si Thammarat, Thailand

Authors’ Email Addresses:

**^*^Corresponding Author**: Manas Kotepui; manas.ko@wu.ac.th

Polrat Wilairatana; polrat.wil@mahidol.ac.th

Praphassorn Mahannop; praphassornmahannop367@gmail.com

Thanita Tussato; thanita.tu@mail.wu.ac.th

I-mee Hayeedoloh; imee.ha@mail.wu.ac.th

Rachasak Boonhok; rachasak.bo@wu.ac.th

Wiyada Kwanhian Klangbud; kwiyada@wu.ac.th

Wanida Mala; wanida.ma@wu.ac.th

Kwuntida Uthaisar Kotepui; kwuntida.ut@wu.ac.th

**Table S2.** Characteristics of the included studies

| **No.** | **Author, year** | **Study location (year)** | **Study design** | **Participants (n)** | **Male %** | **Age (years)** | **Malaria cases (n)** | ***Plasmodium* spp.** | ***CRP (number of cases in each group)** | ****CRP in each group (number of cases in each group):** | **CRP measurement** |
| --- | --- | --- | --- | --- | --- | --- | --- | --- | --- | --- | --- |
| 1 | Amah et al.,2011 | Nigeria | Case control study | Uncomplicated malaria (50), hepatitis B (50), healthy controls (40) | NS | range 30-65 | 50 | *P. falciparum* (50) | 0.78 ± 0.13 mg/dL | Uncomplicated malaria (50): 0.78 ± 0.13 mg/dL,  Hepatitis B (50): 0.71 ± 0.11 mg/dL,  Healthy control (40): 0.32 ± 0.12 mg/dL | Turbidimetric immunoassay |
| 2 | Andrade et al.,2010 | Brazil (2007) | Cross-sectional study | Active and passive malaria case detections (219): asymptomatic malaria (60), uncomplicated malaria (90), severe malaria (19), febrile and healthy controls (90) | 101/169, 59.8%  febrile and healthy controls (90):  male (39), female (51) Asymptomatic malaria: male (30), female (30) Uncomplicated malaria: male (22), female (28) Severe malaria: male (10), female (9) | All groups mean 34.8,  Febrile and healthy controls 38 (range 25-31), asymptomatic 42 (range 32-48.2), uncomplicated malaria 33 (26.7-48), severe malaria 22 (range 16-35) | 129 | *P. vivax* (129) | Asymptomatic malaria (60): 6.6 (4.12-9.35) ng/mL,  Uncomplicated malaria (50): 6.5 (4.9-8.7) ng/mL  Severe malaria (19): 15.3 (11.9-20.6) ng/mL,  Febrile and healthy controls (90): 5.65 (3.7-9.47) ng/mL | Asymptomatic malaria (60): 6.67 ± 1.52 ng/mL,  Uncomplicated malaria (50): 6.65 ± 1.12,  Severe malaria (19): 15.8 ± 2.52 ng/mL,  Febrile and healthy controls (90): 6.12 ± 1.68 ng/mL | NS |
| 3 | Aninagyei et al.,2021 | Ghana (2017-2018) | Prospective observational study | Blood donors (200) | 100%,  Male (200) | range 18–35 | *P. falciparum* (80) | *P. falciparum* (80) | 1.74 ± 0.19 mg/L | Asymptomatic malaria (80): 1.74 ± 0.19 mg/L Healthy blood donor (120): 0.75 ± 0.23 mg/L | Fluorescence Immunoassay |
| 4 | Atkinson et al.,2015 | Kenya (2001-2003) | Cohort study | Children (324) | NS | 0–1 (63), 1–3 (161), 3–5(142), 5–8 (227) | 0–1 year (1 /62), 1–3 years (10/155),3–5 years (21/141),5–8 years (38/224) | *P. falciparum* (70) | 0–1 year (1 /62): 7.05 mg/L,1–3 years (10/155): 2.48 (0.75-8.14) mg/L, 3–5 years (21/141): 3.28 (1.45 -7.44) mg/L, 5–8 years (38/224): 1.47 (1.08, 2.78) mg/L | Asymptomatic malaria (70): 3.07 ± 3.52 mg/L  Healthy control (452): 1.08 ± 0.34 mg/L | Turbidimetric immunoassay |
| 5. | Bhardwaj et al., 2019 | India (2016-2017) | Prospective observational study | Individuals (96):  *P. falciparum* (74), healthy controls (22) |  | All group mean 20.83,  *P. falciparum* 18 (range 13–29.25), healthy controls  28 (range 25.75–40.75) | NS | *P. falciparum* (74) | Severe malaria (7): 2 (1.8-3.9) mg/dL,  Uncomplicated malaria (67): 1.4 (1-2.47) mg/dL,  Healthy controls (22): 0.1 (0.1-0.2) mg/dL | Severe malaria (7): 2.43 ± 0.66 mg/dL  Uncomplicated malaria (64): 1.57 ± 0.47 mg/dL,  Healthy controls (22): 0.13 ± 0.21 mg/dL | Turbidimetric immunoassay |
| 6. | Bruneel et al., 2016 | France (2007-2010) | Prospective observational study | Patients with malaria (295): severe malaria (155), uncomplicated malaria (140) | 204/295, 69.2%  Severe malaria (155): male (104), female (51), uncomplicated malaria (140): male (100), female (40) | Severe malaria (155): 44.4 ± 14, Uncomplicated malaria (140): 39.3 ± 12.5 | 295 | *P. falciparum* (295) | Severe malaria (155): 165.8 ± 80.9 mg/L, Uncomplicated malaria (140): 138.4 ± 82.8 mg/L | Severe malaria (155): 165.8 ± 80.9 mg/L, Uncomplicated malaria (140): 138.4 ± 82.8 mg/L | Turbidimetric immunoassay |
| 7. | Conroy et al.,2011 | Malawi (2001-2006) | Cross-sectional study | Pregnant women (465) | 0%,  Female (465) | Mean 20.5 (range 18.3–25.3) | 139 | *P. falciparum* (139) | Median 60.2 mg/mL  (range16.7–72.7) | Asymptomatic malaria (24): 52.5 ± 16.1 mg/mL,  Healthy control (326): 21.2 ± 9.1  mg/mL | ELISA |
| 8. | Cruz et al.,2019 | Brazil (2006-2007) | Retrospective observational study | Patients from Brazilian Amazon (601): asymptomatic malaria (145), Uncomplicated malaria (179), HBV coinfected (28), HBV patients (29), healthy controls (165), no data available (55) | 179/352, 50.9%  *P. vivax* malaria patients: Asymptomatic malaria: male (63), female (82)  Uncomplicated malaria: male (100), female (79) HBV coinfected: male (16), female (12) | All group mean (asymptomatic and uncomplicated malaria) 38,  Asymptomatic malaria: median 43 (IQR 34–51.25)  Uncomplicated malaria: 32 (IQR 24–47) HBV coinfected: 30.5 (22.5–45.75) | 324 | *P. vivax* (324) | Asymptomatic malaria (127): 7.8 mg/L (4.5–10.3), Uncomplicated malaria (141): 15.5 mg/L (7.8–33.75),  HBV patients (29): 4.8 (4.0–6.75) mg/L | Uncomplicated malaria (141): 18.1 ± 7.5 mg/L, Asymptomatic malaria (127): 7.6 ± 1.69 mg/L, HBV patients (29): 5.09 ± 0.83 mg/L | NS |
| 9. | Cusick et al.,2016 | Ugandan (2010-2013) | Prospective observational study | Children (239):  cerebral malaria (79), severe malarial anemia (77), community controls (83) | 123/239, 51.5%  Cerebral malaria:  male (42), female (37) Severe malarial anemia:  male (46), female (31) Community controls:  male (35), female (48) | All group mean 3,  Cerebral malaria: 3.1 ± 1.0 Severe malarial anemia: 2.8 ± 1.0 Community controls: 3.1± 0.9 | 156 | *P. falciparum* (156) | Cerebral malaria (79): 822.5 mg/L (555.4-1,103.9), Severe malarial anemia (77): 581.3 mg/L (334.6-842.7), healthy control (83): 4.2 mg/L (1.1-15.2) | Cerebral malaria (79): 826.1 ± 158.3 mg/L, Severe anemia (77): 585 ± 146.7 mg/L,  Healthy controls (83): 6.18 ± 4.08 mg/L  Severe malaria (cerebral and severe anemia) (156): 707.1 ± 194.4 mg/L | Immunoassay |
| 10. | Elphinstone et al., 2019 | Malawi (2011-2013) | Cohort study | Pregnant women (1,628) | 0%,  Female (1,628) | Mean (21.3), median 21  (range 18-25) | 1,138 | *P. falciparum* (1,138) | Asymptomatic malaria (643): 5.69 (2.69–13.5) ug/mL,  Healthy controls (813): 2.45 (1.17–5.23) ug/mL | Asymptomatic malaria (643): 6.89 ± 3.14 ug/mL,  Healthy controls (813): 2.83 ± 1.2 ug/mL | ELISA |
| 11. | Eriksson et al., 1989 | Sweden (1975-1986) | Retrospective observational study | Patients with malaria (258) | 182/258, 70.5%  Male (182), female (76) | Mean (32.5), Median 31 (range 1-67) | 258 | *P. falciparum* (86), *P. vivax* (111), *P. ovale* (50), *P. malariae* (1), mixed infections of *P. falciparum*, *P. vivax*, and *P. ovale* (10) | *P. falciparum* (86): 55 mg/L (8-180),  Non-falciparum (172): 75 mg/L (1-180), Febrile control (120): 39 (5-480) mg/L | *P. falciparum* (86): 74.5 ± 49.7 mg/L,  Non-falciparum (172): 82.8 ± 51.7 mg/L, Febrile control (120): 140.8 ± 137.2 mg/L  Uncomplicated malaria (258): 80 ± 51.1 mg/L | Immunoassay |
| 12. | Gjørup et al.,2007 | Denmark | Prospective observational study | Patients with malaria (40): uncomplicated malaria (39), severe malaria (1) | 24/40, 60%  Malaria (40): male (24), female (16) | All age mean 39.6, Malaria (40): 33 (range 19-84),  Febrile controls (66): 38 (range 2-74) | 40 | *P. falciparum* (28), *P. vivax* (11), *P. ovale* (1) | Malaria (40): 1,001 ± 104 mM,  Febrile controls (66): 608 ± 113 mM  *P. falciparum* (28): 1,179 ± 119 mM,  *P. vivax* and *P. ovale* (12): 593 ± 145 mM | Malaria (40*): 1,001 ± 104 mM, Febrile controls (66): 608 ± 113 mM  *One case was a severe case | NS |
| 13. | Gyan et al.,2002 | Ghana (1997) | Prospective observational study | Patients with malaria (158): cerebral malaria (77), severe anemia (28), uncomplicated malaria (53) | NS | Range 0.5-12 | 130 | *P. falciparum* (130) | Cerebral malaria (77): 240 µmol/L (150–340),  Severe anemia (28): 230 µmol/L (172–295),  Uncomplicated malaria (53): 110 µmol/L (77–200) | Cerebral malaria (77): 242.5 ± 54.8 µmol/L, Severe anemia (28): 231.8±35.5 µmol/L,  Uncomplicated malaria (53): 124.3 ± 35.5 µmol/L  Severe malaria (cerebral malaria and severe anemia) (105): 239.6 ± 50.4 µmol/L | ELISA |
| 14. | Hollestelle et al.,2006 | Ghana | Prospective observational study | Children (253) | NS | Range 6 months-6 years | 143: Cerebral malaria (26), Non-cerebral severe malaria (73), Uncomplicated malaria (44) | *P. falciparum* (143) | Cerebral malaria (26): 142.5 ± 120.5 mg/L, Non-cerebral severe malaria (73): 64.8 ± 97.8 mg/L, Uncomplicated malaria (44): 102.9 ± 80.5 mg/L | Uncomplicated malaria (44): 102.9 ± 80.5 mg/L,  Febrile controls (74): 30.7 ± 48.7 mg/L,  Healthy controls (36): 7.7 ± 11.0 mg/L  All severe malaria (cerebral and non-cerebral) (99): 85.2 ± 109.1 mg/L | Turbidimetric immunoassay |
| 15. | Jakobsen et al.,1998 | Gambia (1993-1994) | Prospective observational study | Blood donors (126): uncomplicated malaria (30), asymptomatic malaria (50), healthy controls (46) | 72/125, 57.6%  Uncomplicated malaria: male (14), female (16), Asymptomatic malaria: male (31), female (19),  Healthy controls: male (27), female (19) | All group mean 2.84, Uncomplicated malaria: 3.5 ± 2.1, Asymptomatic malaria: 2.7 ± 1.3,  Healthy controls: 2.5 ± 1.3 | 80 | *P. falciparum* (80) | Uncomplicated malaria (27): 68 mg/mL (33–95), Asymptomatic malaria (32): 11 mg/mL (3–48),  Healthy controls (40): 1 mg/mL (0-2) | Uncomplicated malaria (27): 66 ± 17.9, Asymptomatic malaria (32): 18.3 ± 13 mg/mL,  Healthy controls (40): 1 ± 0.61 mg/mL | ELISA |
| 16. | Klenerman et al., 1992 | United Kingdom | Prospective observational study | Febrile patients (41): | NS | NS | 17 | *P. falciparum* (14), *P. vivax* (3) | Uncomplicated malaria (17): 6.7 ± 8.6 mg/dL, Febrile controls (24): 2.7 ± 3.5 mg/dL | Uncomplicated malaria (17): 6.7 ± 8.6 mg/dL, Febrile controls (24): 2.7 ± 3.5 mg/dL | NS |
| 17. | Kremsner et al.,1996 | Gabon | Prospective observational study | Patients with malaria (93): uncomplicated malaria (64), severe malaria (29) | 55/93, 59.1%  Uncomplicated malaria (64):  Adult: male (8), female (15)  Children: male (26), female (15)  Severe malaria (children) (29): male (21), female (8) | All group mean 13, Uncomplicated malaria: adult: 20 (range 16-64), children: 10 (range 4-15)  Severe malaria (children): 3 (range 0.5-10) | 93 | *P. falciparum* (93) | Uncomplicated malaria (64): adult: 49 ± 40 ug/mL, children: 46 ± 43 ug/mL  Severe malaria (children): 137 ± 71 ug/mL | Severe malaria (29): 137 ± 71 ug/mL  Uncomplicated malaria (64): 47.1 ± 41.7 ug/mL | Radial immunodiffusion |
| 18. | Kung’u et al., 2009 | Tanzania (2003-2004) | Cross-sectional study | Children (2,322) | 1180/2322, 50.8%  Male (1,180), female (1,142) | All group mean 17,  Malaria (79): no malaria (194): 1.41 ± 6 0.59 | 154 | *P. falciparum* (154) | Asymptomatic malaria (140): 9.57 (7.84, 11.7) mg/L,  Healthy controls (393): 3.54 (3.13, 4.01) mg/L | Asymptomatic malaria (140): 9.66 ± 1.12 mg/L,  Healthy controls (393): 3.56 ± 0.33 mg/L | ELISA |
| 19. | Kutsuna et al.,2015 | Japan (2005-2013) | Retrospective observational study | Patients with malaria, dengue, and enteric fever (202): dengue fever (85), malaria (86), enteric fever (31) | 136/202, 67.3%  Dengue fever: male (50), female (35)  Malaria: male (64), female (22) Enteric fever: male (22), female (9) | All group mean 33.1, Dengue fever: 32.8 ± 12.9, malaria: 34.0 ± 11.4, enteric fever: 31.4 ± 11.6 | 86 | *P. falciparum* (56), *P. vivax* (20), *P. ovale* (8),  *P. malariae* (1), *P. knowlesi* (1) | Median 8.1 mg/dL (4.0-13.1) | Uncomplicated malaria (86): 53.4 ± 8 mg/dL,  Dengue (85): 28.5 ± 1.31 mg/dL,  Enteric fever (31): 6.55 ± 2.08 mg/dL,  Dengue and enteric fever (116): 22.6 ± 9.88 mg/dL | NS |
| 20. | Leli et al.,2020 | Italy (2011-2019) | Retrospective observational study | Patients suspected with malaria (211): malaria (21), febrile controls (190) | 127/211, 60.2%  Male (127), female (84) | All group mean 38 Malaria: mean 42, febrile controls: mean 37.5 | 21 | *P. falciparum* (18), *P. ovale* (2),  *P. vivax* (1) | Median 9.7 mg/dL (IQR 5.3-13.9) | Malaria (21): 9.65 ± 2.49 mg/dL,  Febrile controls (190): 3.68 ± 2.23 mg/dL | NS |
| 21. | Mendonça et al., 2013 | Brazil (2006-2007) | Retrospective observational study | Individuals from Brazilian Amazon (530): endemic controls (176), asymptomatic malaria (148), uncomplicated malaria (187)  Severe malaria: survivors (13), deaths (6) | 245/530, 46.2%  Endemic controls: male (72), female (104)  Asymptomatic malaria: male (70), female (78)  Uncomplicated malaria: male (93), female (94)  Severe malaria: survivors: male (7), female (6)  Severe malaria: deaths: male (3), female (3) | Range 5-70 | 354 | *P. vivax* (354) | Asymptomatic malaria: 7.9 mg/L (IQR4.8-12.3), Uncomplicated malaria: 15.5 mg/L (IQR 8.2-32.8), Severe malaria: Survivors: 13.2 mg/L (IQR 6.7-47.5),  Severe malaria: Deaths: 34.4 mg/L (IQR 16.4-50.7) | Asymptomatic malaria (148): 8.23 ± 2.18 mg/L Uncomplicated malaria (187): 18 ± 7.12 mg/L Severe malaria (19): 24.6 ± 12.8 mg/L,  Healthy and febrile control (176): 5.98 ± 1.71 mg/L | NS |
| 22. | Paul et al., 2012 | India (2010) | Prospective observational study | Patients with malaria infections (71):  *P. falciparum* (42),  *P. vivax* (29) | 51/81, 63%  *P. falciparum*: male (31), female (11) *P. vivax*: male (20), female (9) | NS | 71 | *P. falciparum* (42), *P. vivax* (29) | *P. falciparum*: 26.21 ± 18.95 mg/L, *P. vivax*: 34.8 ± 20.84 mg/L  Severe malaria (23): 47.1 ± 19.1 mg/L  Uncomplicated malaria (48): 23.7 ± 16.4 mg/L | Severe malaria (23): 47.1 ± 19.1 mg/L,  Uncomplicated malaria (48): 23.7 ± 16.4 mg/L | Turbidimetric immunoassay |
| 23. | Peto et al., 2016 | Cambodia (2013-2014) | Prospective observational study | Migrants and visitors in communities (2,151) | 420/656, 64%  Asymptomatic malaria (328): male (210), female (118),  Healthy controls (328): male (210), female (118)  *P. falciparum* (40): male (23), female (17),  *P. vivax* (115): male (81), female (34) | All group mean 25.9, Asymptomatic malaria (328): 27.4 ± 17,  Healthy controls (328): 25.6 ± 17  *P. falciparum* (40): 28 ± 18,  *P. vivax* (115): 28 ± 16 | 328 | *P. falciparum* (40), *P. vivax* (115) | Asymptomatic malaria (328): 0.66 mg/L (0.27-1.99),  Healthy controls (328): 0.58 mg/L (0.21-1.4)  *P. falciparum* (40): 0.43 mg/L (0.22–1.13),  *P. vivax* (115): 0.89 mg/L (0.44–3.31) | Asymptomatic malaria (328): 0.9 ± 0.54 mg/L,  Healthy controls (328): 0.69 ± 0.4 mg/L | ELISA |
| 24. | Righi et al.,2016 | Italy (2011-2013) | Prospective observational study | Patients with malaria (30) | 25/30, 83.3%  Severe malaria: male (8), female (1), Uncomplicated malaria: male (17), female (4) | All group mean 41.3, Severe malaria: 42 ± 13.2, Uncomplicated malaria: 41 ± 9.9 | 30 | *P. falciparum* (30) | Severe malaria: 219 ± 99 mg/dL, Uncomplicated malaria: 96 ± 64 mg/dL | Severe malaria (9): 21.9 ± 9.9 mg/dL, Uncomplicated malaria (21): 9.6 ± 6.4 mg/dL | Fluorescence Immunoassay |
| 25. | Saad et al.,2012 | Sudan (2010) | Prospective observational study | Pregnant women (96): Severe malaria (32), uncomplicated malaria (32), healthy controls (32) | 0%,  Female (96) | All group mean 27.3, Severe malaria: 28.5 ± 6.0, Uncomplicated malaria: 27.0 ± 6.4, Healthy controls: 26.3 ± 2.8) | 64 | *P. falciparum* (64) | Severe malaria: 79.0 µg/mL (IQR 36.2–110.5),  Uncomplicated malaria: 63.0 µg/mL (IQR 22.5–81.7), Healthy control: 8.5 µg/mL (5.0–28.2) | Severe malaria (32): 76.2 ± 21.4 µg/mL, Uncomplicated malaria (32): 57.6 ± 17.1 µg/mL,  Healthy control (32): 22.6 ± 6.73 µg/mL | ELISA |
| 26. | Van Santen et al.,2011 | Gabon (2000-2004) | Retrospective observational study | Pregnant women (69):  negative of placental malaria (39), positive of placental malaria (30) | 0%,  Female (69) | All group mean 17.5, Negative of placental malaria: non-anemia 18.0 (range 17.0–19.0), anemia 17.0 (range 16.0–19.3)  Positive of placental malaria:  non-anemia 17.0 (range 16.0–18.0), anemia 17.0 (range 16.8–19.3) | 30 | *P. falciparum* (30) | Malaria  Non-anemia: 25.0 mg/L (11–47.3)  Anemia: 32.0 mg/L (17.8–67.5),  Non malaria  Non-anemia: 12 mg/L (5–31),  Anemia: 17.5mg/L (6–43.3),  Malaria  Non-anemia: < 10 mg/L (4/16)  Anemia: < 10 mg/L (2/14),  Non-malaria  Non-anemia: < 10 mg/L (10/21)  Anemia: < 10 mg/L (8/18) | Asymptomatic malaria (30): 31.9 ± 13.3 mg/L,  Healthy control (39): 18.8 ± 9.57 mg/L | Immunologic agglutination |
| 27. | van Wolfswinkel et al., 2013 | Netherlands  (1999-2012) | Cohort study | Patients with malaria (440): severe *P. falciparum* malaria (61), Uncomplicated *P. falciparum* malaria (259), non-falciparum malaria (120) | 313/440, 71.1%  Severe *P. falciparum* malaria: male (39), female (22),  Uncomplicated *P. falciparum* malaria: male (191), female (68), Non-falciparum malaria: male (83), female (37) | All group mean 41.4, Severe *P. falciparum* malaria: 44 (range 4–70),  Uncomplicated *P. falciparum* malaria: 39 (range 11–78),  Non-falciparum malaria: 36 (range 15–77) | 440 | *P. falciparum* (320), *P. vivax* (88), *P. ovale* (27), *P. malariae* (4), *P. knowlesi* (1) | Severe *P. falciparum* malaria (61): 182 mg/L (65–476) , Uncomplicated *P. falciparum*: 85 mg/L (5–320),   Non-falciparum: 71 mg/L (14–348) | Severe *P. falciparum* malaria (61): 226.3 ± 118.7 mg/L, Uncomplicated *P. falciparum* malaria (259): 123.8 ± 91 mg/L,  Non-falciparum malaria (120): 126 ± 96.4 mg/L | NS |
| 28. | Verhoef et al.,2001 | Kenya (1997) | Cross-sectional study | Children in communities (318) | 173/317, 54.6%  Male (173), female (144) | Mean (19 months), 18.9 months (range 2–36) | 56 | *P. falciparum* (56) | Asymptomatic malaria (15): 12.5 mg/L,  Healthy controls (76): 6.8 mg/L | Asymptomatic malaria (15): 12.5 mg/L,  Healthy controls (76): 6.8 mg/L | Turbidimetric immunoassay |
| 29. | Yayan et al.,2017 | Germany (2004-2012) | Case control study | Patients suspected with malaria (53) | 42/53, 79.2%  Malaria: male (12), female (3) Febrile controls: male (30), female (8) | All group mean 43.9, Malaria: 42.3 ± 16.5), Febrile controls: 44.5 ± 18.6 | 15 | *P. falciparum* (11), *P. ovale* (2), *P. malariae* (1), *P. falciparum* mixed with *P. malaria* (1) | Malaria (15): 76.4 ± 75.9 mg/L,  Febrile controls (38): 34.1 ± 44.6 mg/L | Uncomplicated malaria (15): 76.4 ± 75.9 mg/L,  Febrile controls (38): 34.1 ± 44.6 mg/L | Turbidimetric immunoassay |

Abbreviations: SD, standard deviation; NS, not specified; CRP, C reactive protein; ELISA, enzyme-linked immunosorbent assay

* Mean ± SD or median (range or IQR)

****** Mean ± SD
