# Supplementary material for: C-reactive protein as an early biomarker for malaria infection and monitoring of malaria severity: a meta-analysis
Source: Sci Rep. 2021 Nov 11;11:22033. doi: 10.1038/s41598-021-01556-0 (PMC8585865; doi:10.1038/s41598-021-01556-0)
Supplement: Supplementary file 9 — Supplementary Table S3. [file 41598_2021_1556_MOESM9_ESM.docx]

**C-reactive protein as an early biomarker for malaria infection and monitoring of malaria severity: A meta-analysis**

Polrat Wilairatana^1^, Praphassorn Mahannop^2^, Thanita Tussato^2^, I-mee Hayeedoloh^2^, Rachasak Boonhok, Wiyada Kwanhian Klangbud, Kwuntida Uthaisar Kotepui^2^, Manas Kotepui^2^*

^1^Department of Clinical Tropical Medicine, Faculty of Tropical Medicine, Mahidol University, Bangkok, Thailand

^2^Medical Technology, School of Allied Health Sciences, Walailak University, Tha Sala, Nakhon Si Thammarat, Thailand

Authors’ Email Addresses:

**^*^Corresponding Author**: Manas Kotepui; manas.ko@wu.ac.th

Polrat Wilairatana; polrat.wil@mahidol.ac.th

Praphassorn Mahannop; praphassornmahannop367@gmail.com

Thanita Tussato; thanita.tu@mail.wu.ac.th

I-mee Hayeedoloh; imee.ha@mail.wu.ac.th

Rachasak Boonhok; rachasak.bo@wu.ac.th

Wiyada Kwanhian Klangbud; kwiyada@wu.ac.th

Wanida Mala; wanida.ma@wu.ac.th

Kwuntida Uthaisar Kotepui; kwuntida.ut@wu.ac.th

**Table S3.** Quality of the included studies

| **No.** | **Author, year** | **Selection** | | | | **Compatibility** | **Exposure** | | | **Total score**  **(7 stars)** |
| --- | --- | --- | --- | --- | --- | --- | --- | --- | --- | --- |
|  |  | **Is the case definition adequate?** | **Representativeness of the cases** | **Selection of controls** | **Definition of controls** |  | **Ascertainment of exposure** | **Same method of ascertainment for cases and controls** | **Non-response Rate** |  |
| 1. | Amah et al.,2011 | 🟑 | 🟑 | 🟑 | 🟑 | 🟑🟑 | NA | 🟑 | NA | 7 |
| 2 | Andrade et al.,2010 | 🟑 | 🟑 | 🟑 | 🟑 | 🟑🟑 | NA | 🟑 | NA | 7 |
| 3. | Aninagyei et al.,2021 | 🟑 | 🟑 | 🟑 | 🟑 | 🟑🟑 | NA | 🟑 | NA | 7 |
| 4. | Atkinson et al.,2015 | 🟑 | 🟑 | 🟑 | 🟑 | 🟑🟑 | NA | 🟑 | NA | 7 |
| 5. | Bhardwaj et al., 2019 | 🟑 | 🟑 | 🟑 | 🟑 | 🟑🟑 | NA | 🟑 | NA | 7 |
| 6. | Bruneel et al., 2016 | 🟑 | 🟑 |  | 🟑 | 🟑🟑 | NA | 🟑 | NA | 6 |
| 7. | Conroy et al.,2011 | 🟑 | 🟑 | 🟑 | 🟑 | 🟑🟑 | NA | 🟑 | NA | 7 |
| 8. | Cruz et al.,2019 | 🟑 | 🟑 |  | 🟑 | 🟑🟑 | NA | 🟑 | NA | 6 |
| 9. | Cusick et al.,2016 | 🟑 | 🟑 |  | 🟑 | 🟑🟑 | NA | 🟑 | NA | 6 |
| 10. | Elphinstone et al., 2019 | 🟑 | 🟑 | 🟑 | 🟑 | 🟑🟑 | NA | 🟑 | NA | 7 |
| 11. | Eriksson et al., 1989 | 🟑 | 🟑 |  | 🟑 | 🟑🟑 | NA | 🟑 | NA | 6 |
| 12. | Gjørup et al.,2007 | 🟑 | 🟑 |  | 🟑 | 🟑🟑 | NA | 🟑 | NA | 6 |
| 13. | Gyan et al.,2002 | 🟑 | 🟑 |  | 🟑 | 🟑🟑 | NA | 🟑 | NA | 6 |
| 14. | Hollestelle et al.,2006 | 🟑 | 🟑 | 🟑 | 🟑 | 🟑🟑 | NA | 🟑 | NA | 7 |
| 15. | Jakobsen et al.,1998 | 🟑 | 🟑 | 🟑 | 🟑 | 🟑🟑 | NA | 🟑 | NA | 7 |
| 16. | Klenerman et al., 1992 | 🟑 | 🟑 |  | 🟑 | 🟑🟑 | NA | 🟑 | NA | 6 |
| 17. | Kremsner et al.,1996 | 🟑 | 🟑 |  | 🟑 | 🟑🟑 | NA | 🟑 | NA | 6 |
| 18 | Kung’u et al., 2009 | 🟑 | 🟑 | 🟑 | 🟑 | 🟑🟑 | NA | 🟑 | NA | 7 |
| 19. | Kutsuna et al.,2015 | 🟑 | 🟑 |  | 🟑 | 🟑🟑 | NA | 🟑 | NA | 6 |
| 20. | Leli et al.,2020 | 🟑 | 🟑 |  | 🟑 | 🟑🟑 | NA | 🟑 | NA | 6 |
| 21. | Mendonça et al., 2013 | 🟑 | 🟑 | 🟑 | 🟑 | 🟑🟑 | NA | 🟑 | NA | 7 |
| 22. | Paul et al., 2012 | 🟑 | 🟑 |  | 🟑 | 🟑🟑 | NA | 🟑 | NA | 6 |
| 23. | Peto et al., 2016 | 🟑 | 🟑 | 🟑 | 🟑 | 🟑🟑 | NA | 🟑 | NA | 7 |
| 24. | Righi et al.,2016 | 🟑 | 🟑 |  | 🟑 | 🟑🟑 | NA | 🟑 | NA | 6 |
| 25. | Saad et al.,2012 | 🟑 | 🟑 | 🟑 | 🟑 | 🟑🟑 | NA | 🟑 | NA | 7 |
| 26. | Van Santen et al.,2011 | 🟑 | 🟑 | 🟑 | 🟑 | 🟑🟑 | NA | 🟑 | NA | 7 |
| 27. | van Wolfswinkel et al., 2013 | 🟑 | 🟑 |  | 🟑 | 🟑🟑 | NA | 🟑 | NA | 6 |
| 28. | Verhoef et al.,2001 | 🟑 | 🟑 | 🟑 | 🟑 | 🟑🟑 | NA | 🟑 | NA | 7 |
| 29. | Yayan et al.,2017 | 🟑 | 🟑 |  | 🟑 | 🟑🟑 | NA | 🟑 | NA | 6 |

🟑A star rating, NA: Not assessed
